# Core transcriptional regulatory circuitry identified from patient-derived tumor cells provides a specific vulnerability for rhabdomyosarcoma

**DOI:** 10.1038/s41419-026-08949-x

**Published:** 2026-06-13

**Authors:** Shuangai Liu, Ziqi He, Xue Chen, Yinbing Tang, Min He, Lifeng Zhang, Weili Xu, Jinhu Wang, Ting Tao

**Affiliations:** 1 Department of Surgical Oncology, Children’s Hospital Zhejiang University School of Medicine, National Clinical Research Center for Children and Adolescents’ Health and Diseases, Hangzhou, China; 2 Pediatric Cancer Research Center, National Clinical Research Center for Children and Adolescents’ Health and Diseases, Children’s Hospital Zhejiang University School of Medicine, Hangzhou, China; 3 Department of Pediatric Surgery, The Second Hospital of Hebei Medical University, Shijiazhuang, China; 4 The First Clinical Institute, Zunyi Medical University, Zunyi, China; 5 Zhejiang Key Laboratory of Neonatal Diseases, Hangzhou, China; 6 Cancer Center, Zhejiang University, Hangzhou, China

**Keywords:** Paediatric cancer, Sarcoma

## Abstract

Rhabdomyosarcoma (RMS) is the most common soft tissue sarcoma in children and adolescents, with limited targeted treatment options. Core transcriptional regulatory circuitry (CRC) proteins play crucial roles in tumor cell identity and survival. Here, we established 15 patient-derived tumor cells (PDCs) from RMS specimens and found that these cells retained the expression of RMS hallmark and dependency genes, as well as the genetic mutations in primary tumors. Cleavage under targets and tagmentation sequencing (CUT&Tag-seq) for H3K27ac in these RMS PDCs identified super-enhancers and the associated CRC, which contained SMAD3, RUNX2, JUN and FOS. These factors formed an interconnected auto-regulatory loop that maintained the malignant cell state in RMS. Knocking down each of these genes decreased the expression of all the CRC members and impaired tumor cell growth in RMS. CDK7 inhibition by THZ1 disrupted the CRC and effectively suppressed cell proliferation in RMS. Our study demonstrates a common CRC in RMS that is essential for tumor maintenance, providing a promising therapeutic vulnerability for RMS.

## Introduction

Rhabdomyosarcoma (RMS) is the most common soft tissue sarcoma in children and adolescents, characterized by the malignant transformation of skeletal muscle progenitor cells [1]. RMS presents as a heterogeneous group of tumors, classified into four histological subtypes: embryonal (ERMS), alveolar (ARMS), spindle/sclerosing cell (SRMS), and pleomorphic (PRMS) [2]. These subtypes are associated with distinct molecular alterations. ARMS is frequently characterized by chromosomal translocations generating *PAX3*-*FOXO1* or *PAX7*-*FOXO1* fusion oncoproteins, which define fusion-positive RMS (FP-RMS) and are associated with more aggressive clinical behavior [3]. In contrast, most ERMS cases lack these fusions and are classified as fusion-negative RMS (FN-RMS); they commonly exhibit alterations in RAS pathway genes (e.g., *NRAS*, *KRAS*, *HRAS*), *BCOR*, *NF1*, *TP53*, or other signaling regulators [4–6]. SRMS represents a heterogeneous category, including cases with *MYOD1* mutations [7], while PRMS occurs predominantly in adults and is typically fusion-negative, with a high frequency of *TP53* (79%) and *RB1* (43%) alterations [8]. Although histologic classification and fusion status are strongly correlated—particularly for ARMS and ERMS—molecular classification based on fusion status has become increasingly important for biological stratification and risk assessment. Despite advances in multimodal therapy, the prognosis for patients with metastatic or recurrent disease remains poor [4]. A better understanding of the molecular and genetic mechanisms underlying RMS pathogenesis and progression is crucial for developing effective targeted therapies and improving clinical outcomes.

In many cell types, a small set of core transcription factors dominates the control of cell identity by binding each other’s regulatory elements and forming interconnected autoregulatory loops [9]. Such core transcriptional regulatory circuitry (CRC) factors govern key gene expression programs, and are often driven by large enhancer clusters known as super-enhancers (SE). In cancer, dysregulated CRCs can lock cells into an oncogenic state [10, 11]. It has been reported that PAX3-FOXO1 establishes a myoblastic SE landscape and sets up autoregulatory loops in FP-RMS, in collaboration with the master transcription factors, including MYOG, MYOD, MYCN, and SOX8 [12, 13]. These studies suggest that a specific CRC sustains the malignant cell state in FP-RMS. However, whether a common CRC exists across different RMS subtypes, including FN-RMS, remains to be elucidated.

Patient-derived tumor cells (PDCs) have emerged as powerful preclinical models. Unlike established cell lines, PDCs more faithfully capture the heterogeneity, epigenetic landscape, and molecular features of primary tumors [6, 14]. The use of RMS PDCs for epigenetic profiling will provide a powerful approach to advance our understanding of the transcriptional and regulatory mechanisms underlying RMS pathogenesis. In this study, we identified a common CRC composed of SMAD3, RUNX2, JUN and FOS in RMS using de novo established PDCs. These factors form a highly interconnected auto-regulatory loop that sustains the malignant cell state in RMS. Pharmacological inhibition of CDK7 with THZ1 disrupted the integrity of this CRC and effectively suppressed RMS cell proliferation. Collectively, our findings define a shared CRC essential for RMS maintenance and reveal a previously unrecognized epigenetic vulnerability that may be therapeutically exploitable.

## Results

### Generation and characterization of RMS PDCs

**Fig. 1:**
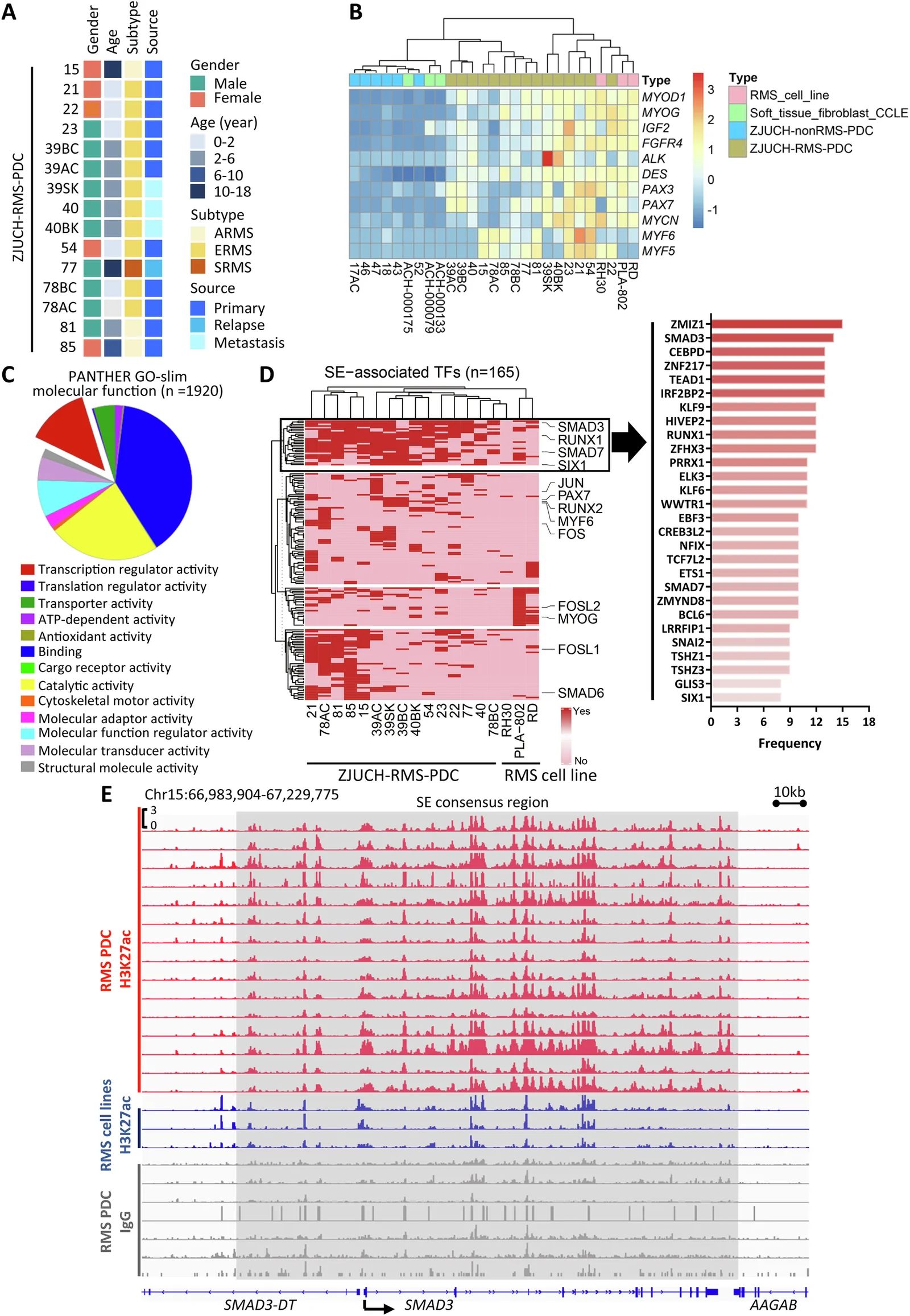
Super-enhance-associated transcription factors identified in patient-derived rhabdomyosarcoma cells. **A** The characteristics of 15 RMS PDCs in ZJUCH-RMS-PDC cohort. **B** Hierarchical clustering of the expression profiles of RMS hallmark genes based on log2 (TPM + 1). The transcriptome data of 3 fibroblast cell lines (RMS stroma origin) were obtained from Cancer Cell Line Encyclopedia (CCLE). TPM, transcripts per kilobase of exon per million mapped reads. Scale bar represents range of the relative expression levels. **C** Classification of 1920 SE-associated genes identified in RMS PDCs (*n* = 15) and cell lines (*n* = 3) by GO-slim molecular function analysis through the PANTHER (Protein ANalysis THrough Evolutionary Relationships) Classification System. GO, gene ontology. **D** Hierarchical clustering of 165 SE-associated transcription factors. The most common subset of transcription factors is indicated with a black box, with their frequencies displayed in the right panel. **E** CUT&Tag-seq tracks for H3K27ac at *SMAD3* gene locus in all 15 RMS PDCs and 3 RMS cell lines. CUT&Tag-seq read densities are plotted as the number of reads per million reads sequenced for each sample. Super-enhancer (SE) consensus region is denoted. IgG is used as control.

We previously established 9 PDCs from RMS specimens, and showed that these PDCs retained the genetic alterations and the expression of RMS hallmark and dependency genes in matched primary tumors, providing valuable tools for preclinical studies [6]. Using the same approach, we generated and validated 6 additional RMS PDCs, which predominantly displayed elongated or spindle-shaped morphology (Fig. S1A; Table S1). The characteristics of all the 15 RMS PDCs (referred to as ZJUCH-RMS-PDC cohort) were summarized in Fig. 1A. All of these RMS PDCs robustly expressed RMS hallmark genes, including *MYOD1*, *MYOG* and *DES*, as compared with the fibroblast-like non-RMS PDCs derived from RMS tissues (Fig. 1B). Further, the majority of the high-confidence mutations, including those in *HRAS*, *NRAS*, *TP53*, *MYOD1*, *TSC2* and *ARID1A*, were well preserved in the RMS PDCs as in their matched tumor tissues (Fig. S1B; Table S2). Thus, our RMS PDCs serve as unique and faithful models that recapitulate the genetic and transcriptomic features of their primary tumors, making them valuable tools for preclinical studies.

### SE-associated transcription factors form an interconnected transcriptional network in RMS

To identify SEs and the associated CRC of RMS, we performed cleavage under targets and tagmentation sequencing (CUT&Tag-seq) for H3K27ac in all 15 RMS PDCs, as well as in 3 RMS cell lines, including RD, RH30 and PLA-802. A median of 19342.5 H3K27ac peaks per sample was identified across all the samples, with 32.23–57.06% located in promoters within 3 kilobases (kb) from the transcription start site (TSS), 28.15–44.16% in introns, and 10.16–16.79% in distal intergenic regions (Fig. S1C). SEs were identified using the ROSE algorithm (see “Materials and methods” for detail). The SEs were assigned to genes with the nearest TSS, and only those genes present in at least 2 of the 18 RMS cells were selected for further analysis. Gene ontology (GO) analysis with Enrichr [15] revealed that the 1920 SE-associated genes are significantly enriched for functions related to DNA-binding and regulation of DNA-templated transcription (Fig. S1D), indicating their involvement in the transcriptional machinery. Classification of these SE-associated genes by GO-slim molecular function further identified 165 transcription factors (Fig. 1C; Table S3). Unsupervised clustering analysis of these SE-associated transcription factors revealed a subset of highly shared factors, including ZMIZ1, SMAD family proteins and RUNX family proteins (Fig. 1D, E). In addition, a group of genes previously reported to be associated with SEs [12, 13, 16], such as CEBPD, ZNF217, PRRX1, SIX1 and KLF family proteins (Fig. 1D), were also identified, indicating the reproducibility of the underlying epigenetics data.

**Fig. 2:**
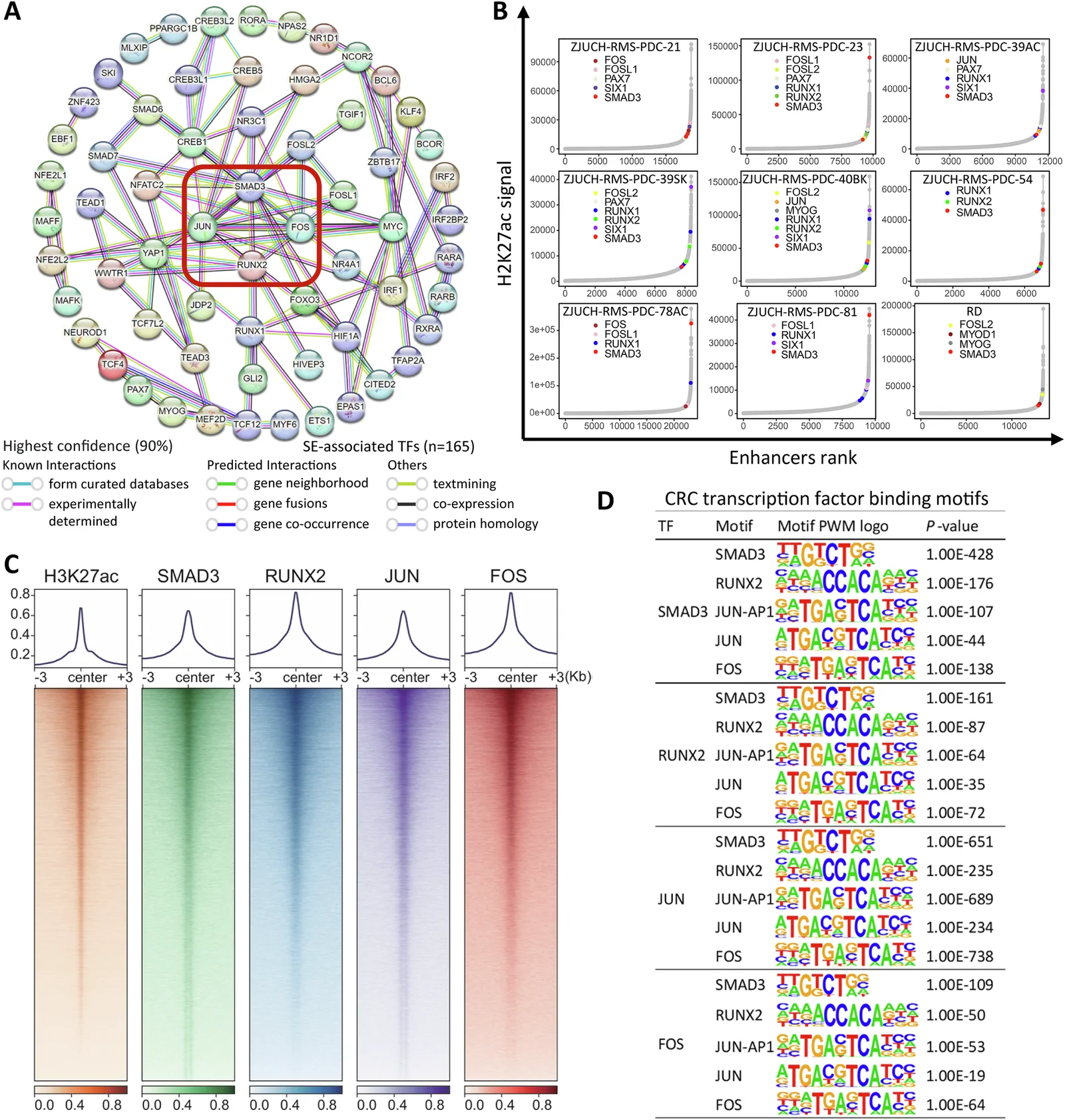
SMAD3, RUNX2, JUN and FOS bind consensus DNA sequences in a coordinated manner. **A** Transcriptional regulatory network analysis of 165 super-enhancer (SE)-associated transcription factors (TFs) identified from RMS with the STRING database. The minimum interaction score was set to the highest confidence level (90%). The core regulatory node is highlighted with a red box, and the methods used to identify the protein-protein interactions are indicated by the line colors. **B** Examples of enhancers ranked by H3K27ac signal in RMS PDCs and cell lines as indicated. Highlighted are SE-associated TFs, including putative CRC members and known TFs such as PAX7, SIX1, MYOG and MYOD1. **C** Metagenes and heatmaps of genome-wide co-occupancy for H3K27ac, SMAD3, RUNX2, JUN and FOS as determined by CUT&Tag-seq in RD cells. Regions (rows) were defined as those enriched in CUT&Tag-seq reads for at least one factor and ranked by the average signal therein. Color keys indicating signals normalized to reads per million are displayed below each heatmap. Kb, kilobase. **D** The HOMER program was used to create position weight matrices (PWM) depicting DNA-binding motifs for each putative CRC member in RD cells. Statistical enrichment of peaks for each motif is denoted by *P*-value.

To identify the core regulatory nodes involving in the pathogenesis of RMS, we performed transcriptional regulatory network analysis with the STRING database [17]. The analysis identified a set of highly interconnected transcription factors based on known and predicted protein-protein interactions, with the minimum interaction score set to the highest confidence level (90%). A core regulatory node was determined, comprising SMAD3, RUNX2, JUN and FOS (Fig. 2A). These factors and their family members displayed strong H3K27ac signal across the majority of samples (Fig. 2B). Correlation analysis of RNA-expression data from The Cancer Genome Atlas (TCGA) using the TIMER2.0 program [18] revealed strong positive correlations among these factors across multiple tumor types (Fig. S2A). Complementary analysis using CRCMapper [11] across all the 18 RMS samples revealed heterogeneity in CRC composition, with SMAD3 recurrently identified as a central component across the majority of samples (Table S4). Thus, we hypothesized that SMAD3, RUNX2, JUN and FOS formed an interconnected auto-regulatory CRC that maintained the malignant cell state in RMS. To test this hypothesis, we performed CUT&Tag-seq for SMAD3, RUNX2, JUN and FOS in RD cells. The results showed that the genomic regions occupied by these putative CRC members were enriched at promoters, introns and distal intergenic regions, exhibiting similar distribution patterns (Fig. S2B). Further, SMAD3, RUNX2, JUN and FOS bind coordinately with each other across the genomic regions marked by H3K27ac (Fig. 2C). By mining the consensus transcription factors from the ChIP-X Enrichment Analysis database, we found that SMAD3, RUNX2, JUN and FOS were among the major factors with target genes overlapping with each other (Fig. S2C). Motif analysis using the HOMER program revealed that the binding motifs for each putative CRC member were enriched within CUT&Tag-seq regions bound by the other members (Fig. 2D). Taken together, these data demonstrate that SMAD3, RUNX2, JUN and FOS bind consensus DNA sequences coordinately with each other, forming an interconnected transcriptional network that may function as a CRC in tumor cells.

### CRC comprising SMAD3, RUNX2, JUN and FOS is required for RMS cell survival

**Fig. 3:**
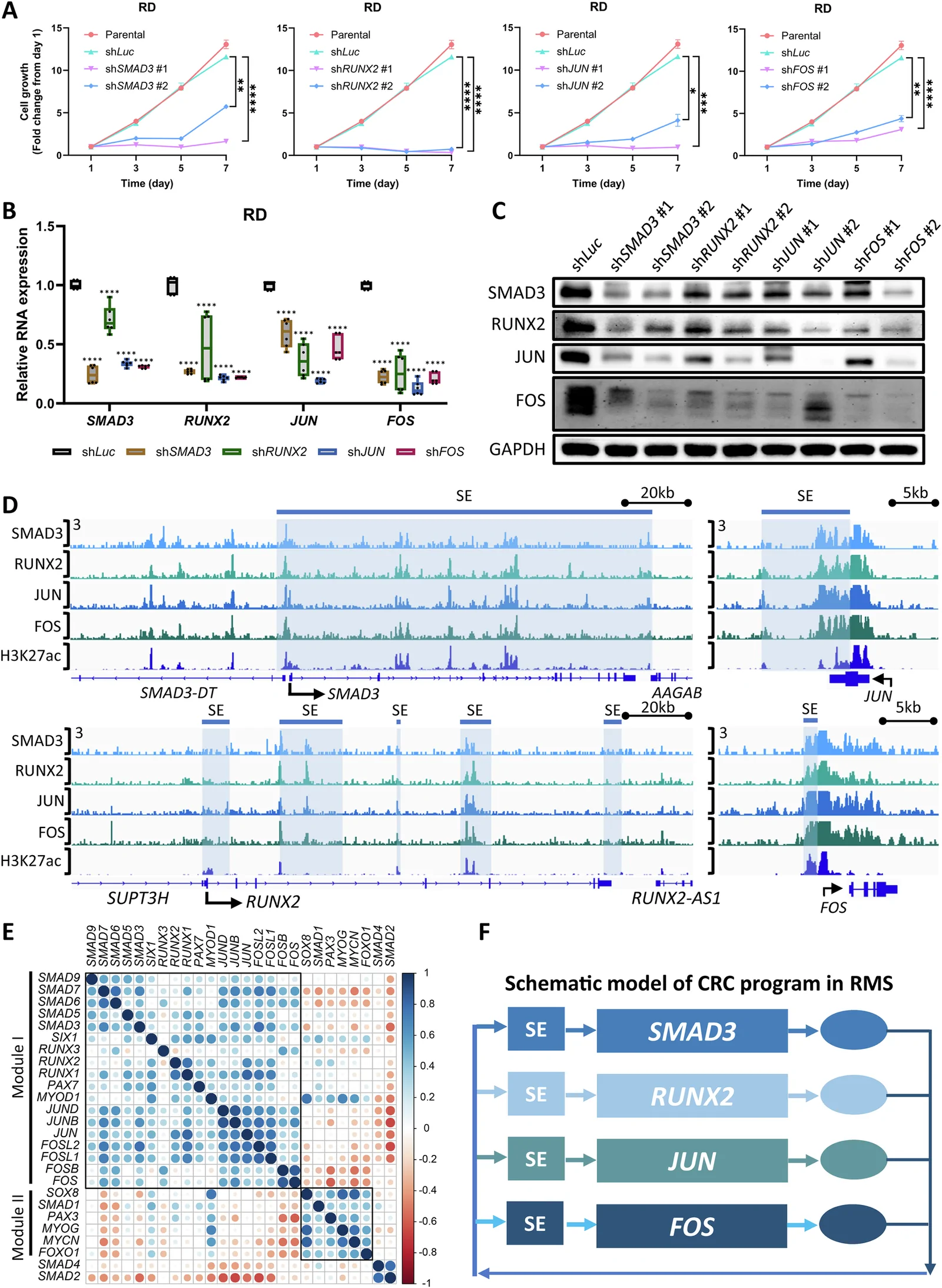
Core transcriptional regulatory circuitry in rhabdomyosarcoma is required for cell survival. RD cells were untransfected (Parental), or transfected with control sh*Luc* or with two independent shRNAs targeting *SMAD3*, *RUNX2*, *JUN* or *FOS*. **A** Relative cell growth at days 1, 3, 5 and 7 was evaluated. Values are means ± SEM of triplicate experiments. Mean values were compared with two-way ANOVA by Dunnett’s test. *P < 0.05, **P < 0.01, ***P < 0.001, ****P < 0.0001. **B** The mRNA expression levels of *SMAD3*, *RUNX2*, *JUN* and *FOS* were detected by quantitative RT-PCR. Values were normalized to *GAPDH*, boxes indicate the median (horizontal line), 25th percentile and 75th percentile; Whiskers indicate the minimum to the maximum. Values were compared with one-way ANOVA by Dunnett’s test. ****P < 0.0001. **C** The protein levels of SMAD3, RUNX2, JUN and FOS were detected by western blot analysis; GAPDH was used as a loading control. **D** CUT&Tag-seq tracks for SMAD3, RUNX2, JUN, FOS and H3K27ac at each CRC gene locus in RD cells. CUT&Tag-seq read densities are plotted as the number of reads per million reads sequenced for each sample. Super-enhancer (SE) regions are denoted. **E** A heatmap of Spearman correlation analysis based on log_2_ (TPM + 1) of core transcription factors from RNA-seq visualizing the correlation coefficients (*r*) between samples. Samples used for plotting include 15 RMS PDCs and 23 RMS cell lines (3 from this study and 20 from the Cancer Cell Line Encyclopedia). **F** SMAD3, RUNX2, JUN and FOS form an interconnected auto**-**regulatory feed-forward loop. Super-enhancers (SEs) and gene loci are denoted by rectangles, and proteins are denoted by oval symbols.

To further investigate the functional role of the transcriptional network involving SMAD3, RUNX2, JUN and FOS, we performed knockdown experiments and phenotypic assessments for each gene. Two independent shRNAs (#1 and #2) targeting each putative CRC member were designed and efficiently reduced the expression of the corresponding gene at both RNA and protein levels in RD cells, while a control shRNA against *luciferase* (sh*Luc*) did not alter the expression levels of each gene (Figs. S3A, B and S8). Knockdown of each putative CRC gene effectively inhibited the growth of ERMS RD cells (Fig. 3A) and reduced the expression of the other CRC members at both the RNA and protein levels (Figs. 3B, C and S9), indicating coordinated regulation among these factors. In addition, knockdown of each of these genes similarly impaired cell growth (Fig. S3C) and decreased the expression of the other CRC components (Figs. S3D, E and S10) in the ARMS cell line RH30, supporting that this regulatory circuit is conserved across both fusion-negative and fusion-positive RMS cells. Further analysis of the CUT&Tag-seq data in the RD cells revealed that SMAD3, RUNX2, JUN and FOS co-occupy consensus genomic regions within their own enhancers (Fig. 3D), suggesting the presence of an auto-regulatory loop that reinforces their coordinated expression. Spearman correlation based on the expression of these factors, their family members and known core transcription factors in 15 RMS PDCs and 22 RMS cell lines (including three from this study and 19 from the Cancer Cell Line Encyclopedia) identified two major modules with an extended regulatory network (Fig. 3E; Table S5). Module I represented a common CRC in RMS, which mainly included SMAD3, RUNX2, JUN, FOS and their family members. Module II, which included PAX3, FOXO1, MYOG, MYCN and SOX8, reflected the specific CRC in FP-RMS as previously reported [12, 13]. The members of these two modules showed partial positive correlation at the expression levels (e.g., *MYOD1* and *SIX1* in module I with the members in module II), indicating a possible interplay between them in FP-RMS. Thus, our data suggest that SMAD3, RUNX2, JUN and FOS form an interconnected auto-regulatory feed-forward CRC, in which they coordinately bind consensus DNA sequences, including within their own enhancer regions, to collectively regulate the expression of their own genes and downstream targets (Fig. 3F).

We next examined the occupancy of SMAD3, RUNX2, JUN, and FOS across SEs, typical enhancers (TEs), and active promoter regions (Fig. S4A). Notably, co-occupancy by multiple CRC factors was markedly enriched at SEs. Specifically, 17.24% of SEs were co-occupied by all four factors, compared to 11.80% at TEs and only 2.24% at promoters. Similarly, higher-order co-occupancy ( ≥ 3 factors) was more frequent at SEs (33.72%) than at TEs (25.99%) and promoters (6.89%). In contrast, regions lacking CRC occupancy were less frequent at SEs (31.80%) compared to TEs (35.72%) and promoters (61.17%). These results demonstrate that SMAD3, RUNX2, JUN, and FOS preferentially co-occupy SE-associated regions, consistent with the canonical features of core regulatory circuitry [19]. We then evaluated the expression of genes associated with each CRC member, stratified by enhancer activity. For genes bound by each CRC member, those associated with SEs were expressed at significantly higher levels than those associated with TEs (Fig. S4B). Furthermore, all genes (Fig. S4C) or SE-associated genes (Fig. S4D) occupied by any of the four CRC members exhibited significantly higher expression compared to those lacking CRC binding. GO analysis revealed that genes co-bound by these four CRC members are significantly enriched for functions related to myogenesis, and are also implicated in RUNX and SAMD-associated pathways (Fig. S4E). Consistently, gene set enrichment analysis (GSEA) showed that knocking down each of the CRC members led to repression of the RUNX and SMAD pathway (Fig. S5), suggesting a functional convergence whereby these factors co-regulate transcriptional programs critical for RMS pathogenesis.

### Pharmacological inhibition of the CRC suppresses RMS growth

**Fig. 4:**
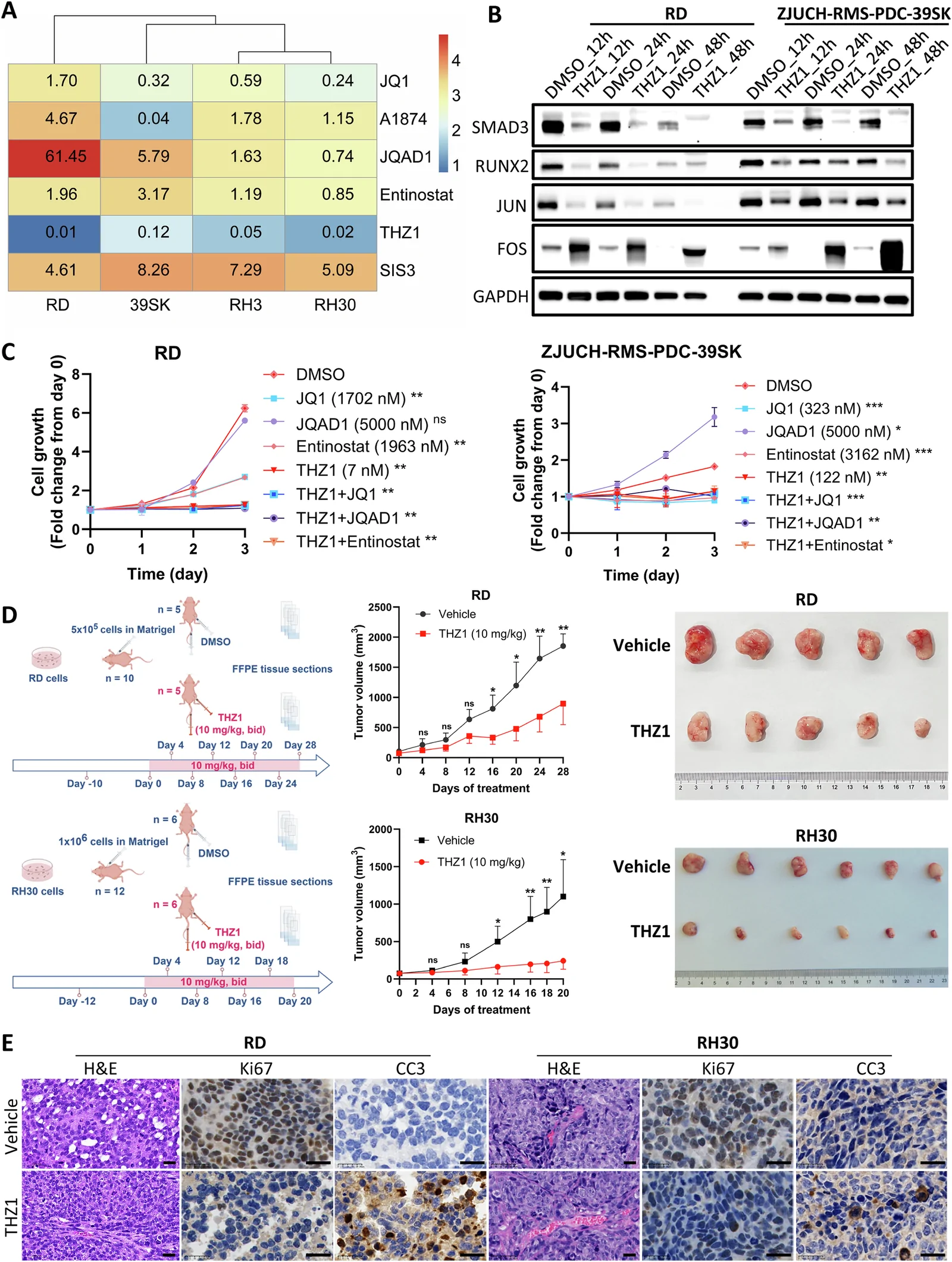
Pharmacological disruption of core transcriptional regulatory circuitry suppresses rhabdomyosarcoma growth. **A** A clustered heatmap showing drug response profile based on log_10_ (IC_50_) of agents targeting epigenetic program. The IC_50_ (μM) of each drug was denoted. **B** RD or ZJUCH-RMS-PDC-39SK cells were treated with vehicle (DMSO) or THZ1 (7 nM for RD, 122 nM for ZJUCH-RMS-PDC-39SK) for 12, 24 or 48 h. The protein levels of SMAD3, RUNX2, JUN and FOS were detected by western blot analysis; GAPDH was used as a loading control. **C** RD or ZJUCH-RMS-PDC-39SK cells were treated with vehicle (DMSO) or different drug combinations. Relative cell growth at days 1, 2 and 3 was evaluated. Values are means ± SEM of triplicate experiments. Mean values were compared with two-way ANOVA by Dunnett’s test. *P < 0.05, **P < 0.01, ***P < 0.001, ns, not significant. The IC_50_ values and statistical significance (comparison of drug versus DMSO) are noted after each drug name. **D** Mice were injected subcutaneously with RD or RH30 cells. Upon tumor establishment, vehicle (DMSO) or THZ1 (10 mg/kg) was administered twice daily starting from day 0, and tumor size was determined every 4 days thereafter. Tumor volumes were analyzed and presented as means ± SD. Values were compared with the Holm-Sidak *t*-test. *P < 0.05, **P < 0.01, ns, not significant. Tumor size was assessed for each group at the experimental endpoint. FFPE, formalin-fixed paraffin-embedded. The schematic diagrams of mouse xenograft tumor experiments were created with FigDraw. **E** Immunohistochemical analysis of morphology (hematoxylin & eosin, H&E), cell proliferation (Ki-67) and apoptosis (cleaved caspase-3, CC3) in RD or RH30 xenograft tumor sections harvested from mice treated with either vehicle (DMSO) or THZ1. Scale bar, 25 μm.

To disrupt the function of CRC in RMS, we pharmacologically targeted the epigenetic transcriptional program using inhibitors against histone readers (BRD4, inhibited by JQ1 or A1874), writers (EP300, inhibited by JQAD1), and erasers (HDAC, inhibited by Entinostat), as well as CDK7 (inhibited by THZ1), a widely expressed regulator of transcription and cell-cycle transition. Among these agents, THZ1 showed the strongest growth-inhibitory effects across a panel of RMS cell lines and PDCs, with the lowest half-maximal inhibitory concentration (IC_50_) of approximately 10 nM (Figs. 4A and S6A). Consistent with its role in regulating transcription, THZ1 treatment markedly reduced the expression of CRC components at both the protein and RNA levels, with the exception of FOS, possibly due to a potential off-target effect of THZ1 or an alternative regulatory mechanism (Figs. 4B and S6B; S11). To more directly assess the contribution of individual CRC factors, we inhibited SMAD3 using the selective inhibitor SIS3. SIS3 treatment significantly impaired cell growth across multiple RMS cell lines and PDCs, with IC₅₀ values ranging from 4.61 to 8.26 μM (Figs. 4A and S6A), and led to a consistent reduction in the expression of the other CRC components at both the RNA and protein levels (Figs. S6C, D and S12).

To evaluate the therapeutic potential of targeting CRC-associated transcriptional programs in RMS, we further assessed a series of drug combinations in RD and ZJUCH-RMS-PDC-39SK cells. Notably, THZ1 monotherapy was sufficient to achieve robust growth inhibition, highlighting its potency as a single-agent treatment strategy in RMS (Fig. 4C). We next established xenograft models using RD (fusion-negative) and RH30 (fusion-positive) cells. Upon tumor establishment, BALB/c nude mice were administered either vehicle (DMSO) or THZ1 (10 mg/kg) twice daily (Fig. 4D, left panel). THZ1 treatment led to a significant reduction in tumor growth compared to the vehicle group (Fig. 4D, middle panel), while the body weights of the mice remained largely unchanged over time and comparable between these two groups (Fig. S7A). At the experimental endpoint, tumors in the THZ1-treated mice were markedly smaller in both size (Fig. 4D, right panel) and weight (Fig. S7B), highlighting the in vivo efficacy of THZ1 in suppressing RMS progression. Tumor tissues were excised and subjected to hematoxylin & eosin (H&E) staining and immunohistochemical analysis. Tumors from vehicle-treated mice exhibited classical histological features of human RMS, characterized by densely packed, poorly differentiated small round blue cells with nuclear atypia and high mitotic activity. In contrast, tumors from THZ1-treated mice exhibited relatively looser cellular organization, more clearly preserved vascular structures, slightly reduced nuclear pleomorphism, decreased cell proliferation and increased cell apoptosis (Fig. 4E). Collectively, these results demonstrate that THZ1 effectively inhibits RMS growth in vitro and in vivo and suppresses the expression of CRC members. While CDK7 inhibition may affect broader transcriptional programs, these findings are consistent with a model in which RMS cells exhibit transcriptional dependencies that include CRC-associated regulatory networks.

## Discussion

Our identification of a common CRC centered on SMAD3, RUNX2, JUN, and FOS highlights a shared transcriptional network in RMS that is crucial for tumor cell survival, representing a promising therapeutic vulnerability in RMS. Using PDC models that faithfully capture the genetic and expression profiles of matched primary tumors [6], we found that these factors formed an interconnected feedforward loop essential for RMS viability. Importantly, this circuitry is distinct from, yet interconnected with, the well‑characterized PAX3-FOXO1-driven CRC in FP-RMS. In FP-RMS, the PAX3-FOXO1 fusion protein establishes a myogenic SE network in concert with MYOD1, MYOG, MYCN, and cofactor SOX8 [12, 13]. Our correlation analysis reflected both the common CRC and PAX3-FOXO1-driven CRC modules, as well as their extended network in RMS, with potential crosstalk between these regulatory architectures. Such interplay may reflect a hierarchical or compensatory relationship between the common and fusion-driven CRCs, which could contribute to the lineage plasticity and aggressive clinical behavior of RMS. Further investigations will be required to delineate the mechanistic basis and functional consequences of this interplay, including whether these CRC modules act in synergy to sustain oncogenic transcriptional programs or confer subtype-specific vulnerabilities that may be exploited therapeutically.

The factors comprising the SMAD3-RUNX2-JUN-FOS circuitry are not unique to RMS. Core transcription factor networks driven by AP-1 and developmental regulators have emerged in diverse pediatric tumors and adult sarcomas. In neuroblastoma, for instance, subset-specific CRCs have been described: a neural crest cell (NCC)-like module contains AP-1 transcription factors (JUN and FOS family members) that operate in NCC-like neuroblastoma cells [20]. Similarly, WNT-subtype medulloblastoma features RUNX2 among its core transcription factors [21]. In adult liposarcoma, the prominence of AP-1/RUNX circuitry is also evident. For example, enhancer profiling in dedifferentiated liposarcoma identified FOSL2 (an AP-1 component), RUNX1, and MYC as CRC factors [22], and genomic amplification of JUN (another AP-1 gene) is common in this disease [23]. SMAD3 has been shown to physically interact with RUNX2 and JUN family proteins to co-regulate target genes in breast cancer [24], implying that TGF-β/SMAD signaling may converge on the RUNX2-AP-1 network. By analogy, the SMAD3-RUNX2-AP-1 module in RMS may reflect shared lineage plasticity programs seen across sarcomas, where dysregulated developmental transcription factors enforce a de-differentiated state. This suggests that RMS may exploit an evolutionarily conserved network of master regulators, integrating TGF-β/SMAD signaling (via SMAD3), osteogenic transcription (via RUNX2), and AP-1 activity (via JUN/FOS) to sustain its oncogenic program.

Lastly, the RMS PDCs established in this study, which accurately preserve the cellular heterogeneity and molecular architecture of their primary counterparts, provide a robust platform for mechanistic studies and preclinical evaluation of precision therapeutic strategies in RMS.

## Materials and methods

### Patient-derived tumor cell (PDC) of rhabdomyosarcoma (RMS)

The method for generating RMS PDCs was as described previously [6]. Briefly, RMS tissues were obtained from post-surgical resection at the Department of Surgical Oncology, Children’s Hospital, Zhejiang University School of Medicine. Histopathological features of these tumors were evaluated by the Department of Pathology, Children’s Hospital, Zhejiang University School of Medicine. After removing blood vessels and necrotic tissues, the remaining tissues were delicately dissected into small fragments and subjected to tissue culture with DMEM/F-12 (1:1) medium (GIBCO) supplemented with 15% fetal bovine serum (ExCell) and 1X penicillin–streptomycin-amphotericin B (Solarbio). The absence of mycoplasma contamination was confirmed by MycoBlue Mycoplasma Detector (Vazyme #D101). This study was approved by the Ethics Committee of Children’s Hospital, Zhejiang University School of Medicine (2020-IRB-049 and 2024-IRB-0191-P-01). All methods were performed in accordance with the relevant guidelines and regulations. All data were acquired with informed consent from patients, their parents or guardians. All results were reported with arbitrary sample ID numbers without linked identifiers.

### RMS cell lines

RMS cell line RD was purchased from the National Collection of Authenticated Cell Cultures (NCACC, Shanghai, China), RH30 from the American Type Culture Collection (ATCC, USA), and PLA-802 as a laboratory stock. RH3 was kindly provided by Dr. Peter Houghton. These cells were maintained in DMEM medium (GIBCO) supplemented with 10% fetal bovine serum (Vazyme) and 1X penicillin-streptomycin-amphotericin B (Solarbio). The absence of mycoplasma contamination was confirmed by MycoBlue Mycoplasma Detector (Vazyme #D101).

### Mouse experiments

Female BALB/c nude mice (6–8 weeks old) were purchased from SLAC Laboratory Animal Co., Ltd. and housed in the Zhejiang Chinese Medical University Laboratory Animal Research Center. All the mouse experiments were performed according to guidelines approved by the Institutional Animal Care and Use Committee of Zhejiang Chinese Medical University under the protocol number IACUC-20250421-05. All methods were performed in accordance with the relevant guidelines and regulations. To evaluate the effect of THZ1 on RMS progression in vivo, RMS cells were suspended in a solution containing 70% DMEM medium (GIBCO) and 30% Matrigel matrix (Corning #354248). Mice were subcutaneously injected with 0.5–1 × 10^6^ cells per animal. When palpable tumors in each group achieved 5–7 mm in diameter (10-12 days), mice were randomized into two groups. Vehicle (DMSO) or THZ1 (10 mg/kg bid) was administered via intraperitoneal injection in the morning and via tail vein injection in the evening. Tumor size was determined every 4 days using the formula V = 0.5 × L × W^2^, where L and W represent the long and short tumor axes, respectively. At the end of the experiments, mice were euthanized, and tumor tissues were harvested for histological analysis. Sample sizes were not predetermined by statistical methods, and no blinding was performed for group allocation.

### Short hairpin RNA (shRNA) knockdown experiments

To knock down *SMAD3*, *RUNX2*, *JUN* or *FOS* in RMS cells, two independent shRNAs (#1 and #2) were designed for each gene. The pLKO.1-TRC lentivirus expression vector containing each shRNA of CRC genes or control sh*Luc* [25] was cotransfected with pMD2.G and psPAX2 into HEK293T cells using the PolyJet™ In Vitro DNA Transfection Reagent (SignaGen #SL100688). Cell culture medium containing the lentivirus was filtered by a 0.45 μm nitrocellulose membrane and infected with RMS cells in the presence of 8 μg/ml polybrene. The cells were treated with puromycin (Beyotime #ST551) at 48 h postinfection to eliminate non-infected cells. The sequences of shRNAs are listed in Table S6.

### RNA extraction and quantitative RT-PCR (qRT-PCR)

Total RNA from RMS cells was extracted using the RNA Purification Kit (ESScience #RN001). For qRT-PCR, cDNA was synthesized using the HiScript III 1st Strand cDNA Synthesis Kit for qPCR (Vazyme #R321). qRT-PCR was performed on a CFX Opus 384 Real-Time PCR System (Bio-Rad) using ChamQ SYBR qPCR Master Mix (Vazyme #Q341) according to the manufacturer’s instructions. Expression data were normalized to the expression of *GAPDH*. Primer pairs for amplification are listed in Table S6.

### Protein extraction and Western blot analysis

Whole-cell lysates were prepared using SDS protein lysis buffer. Western blotting was performed as described previously [26]. Protein samples were separated by a 12% polyacrylamide gel and subsequently transferred to a PVDF membrane (Merck #ISEQ00010). The membrane was blocked with 5% non-fat milk in phosphate-buffered saline/0.1% Tween-20 (PBST) for 1 h at room temperature and then incubated with a primary antibody for 2 h diluted in blocking buffer. Following washes, the membrane was incubated with a horseradish peroxidase (HRP)-conjugated secondary antibody. The results were visualized using the SuperSignal West Pico Chemiluminescent Substrate (ThermoFisher #34577) on a ChemiDoc Imaging System (Bio-Rad). The anti-SMAD3 (rabbit polyclonal, #9523), anti-RUNX2 (rabbit polyclonal, #12556), and anti-FOS (rabbit polyclonal, #2250) antibodies were purchased from Cell Signaling Technology, and anti-JUN (rabbit polyclonal, #ET1608-3), anti-GAPDH (rabbit polyclonal, #ET1601-4), and HRP-conjugated goat anti-rabbit IgG (#HA1001) antibodies from HUABIO.

### RNA sequencing (RNA-seq) and data analysis

RNA-seq and data analysis were performed as previously described [6]. Total RNA from RMS primary tumors and PDCs was extracted by the TRIzol reagent (Thermo Fisher Scientific #15596018CN). Samples were subjected to library preparation for Illumina NovaSeq 6000 S4 sequencing with a 150 bp paired-end sequencing strategy at Zhejiang Biosan Biochemical Technologies Co., Ltd. RNA-seq data were aligned to the GRCh38 reference genome with STAR [27], and reads mapped to each gene were counted by featureCounts [28]. Differentially expressed genes were called by DESeq2 [29], and normalized gene expression values for each sample were obtained from DESeq2 using the variance-stabilizing transformation of the raw counts.

Gene mutations were identified from RNA-seq data through CTAT-Mutations, a machine learning-based RNA-seq variant calling pipeline (https://github.com/NCIP/ctat-mutations). Variants were annotated with ANNOVAR [30], and high-confidence variants were identified through the following steps. First, variants were excluded if they met any of the following criteria: (1) variants not located in exonic or splicing regions, or were synonymous SNVs located in exonic region, or located in repetitive regions of the genome; (2) variants with allele reads less than 3, or sequencing depth less than 8; (3) variant allele frequency (VAF) < 0.1 or allele frequency greater than 0.01 in ExAC or 1000 Genomes database. The candidate variants were selected from the retained variants if they met any of the following criteria: (1) variants were annotated as “pathogenic, “likely pathogenic”, “affects”, “confers_sensitivity”, “association”, “drug response” or “risk factor” in ClinVar or InterVar databases [31, 32], or presented in the COSMIC cancer gene census v97 database [33]; (2) variants not predicted to be unharmful by all of the following programmes: SIFT, Polyphen2_HVAR, Polyphen2_HDIV, LRT, MutationTaster, MutationAssessor, FATHMM and ClinPred. The candidate variants were further annotated with PeCanPIE [34], and only variants classified with a medal were considered as high-confidence variants. Variants were visualized using the R package maftools [35].

### Gene set enrichment analysis (GSEA) and gene ontology (GO) term analysis

The variance-stabilizing transformed gene expression data from DESeq2 were used for GSEA. Genes from the whole transcriptome were ranked according to the statistics from DESeq2. The pre-ranked GSEA option was applied with 1000 permutations for statistical assessment [36]. GSEA was performed using gene signatures from the Molecular Signature Database (MSigDB, v2023.2.Hs). The GO term analysis was performed using the Enrichr program (http://amp.pharm.mssm.edu/Enrichr/) [15], and the top 10 GO terms ranked by *P*-value were visualized.

### Cleavage under targets and tagmentation sequencing (CUT&Tag-seq)

CUT&Tag was performed with NovoNGS CUT&Tag 4.0 High-Sensitivity Kit (for Illumina) (Novoprotein #N259-YH01) according to the manufacturer’s protocol. Briefly, cells were immobilized on ConA magnetic beads and incubated sequentially with primary and secondary antibodies. A pA/G-Tn5 transposome was then applied to target the antibody-labeled chromatin regions, simultaneously cleaving and tagging DNA at these sites. After tagmentation, DNA was extracted using tagment DNA extract magnetic beads, PCR-amplified, purified, and subjected to library preparation for Illumina NovaSeq 6000 S4 sequencing with a 150 bp paired-end sequencing strategy at Annoroad Gene Technology Co., Ltd. The anti-H3K27ac (rabbit polyclonal, #ab4729) and goat anti-rabbit IgG H&L (#ab6702) antibodies were purchased from Abcam; anti-SMAD3 (rabbit polyclonal, #9523), anti-RUNX2 (rabbit polyclonal, #12556), anti-FOS (rabbit polyclonal, #2250) antibodies and normal rabbit IgG (#2729) from Cell Signaling Technology; and anti-JUN (rabbit polyclonal, #ET1608-3) antibody from HUABIO.

### CUT&Tag-seq data analysis and display

CUT&Tag-seq reads were aligned to the GRCh38 reference genome using Bowtie2 [37] with parameters “–local –very-sensitive-local –no-unal –no-mixed –no-discordant -I 10 -X 700”. Peaks were called against IgG control using MACS2 [38] with default parameters at *P* = 1e-9. CUT&Tag-seq peaks were annotated by the R Bioconductor package ChIPseeker [39]. Genes with peaks closest to the transcription start site (TSS) were considered target/binding genes. Active promoters were operationally defined as regions within ±2 kb of TSS marked by H3K27ac, representing promoter-proximal active chromatin. Super-enhancer (SE) in RMS cells was identified using ROSE (https://github.com/stjude/ROSE) as described previously [40, 41]. Briefly, H3K27ac peaks were called twice using MACS2 with parameter sets –keep-dup=auto -p 1e-9 and –keep-dup=all -p 1e-9 and then collapsed together. The collapsed union of regions was used as input for ROSE with parameters -s 12500 -t 1000. H3K27ac peaks located within 12,500 bp of each other were computationally stitched into broader regions, excluding any peaks entirely within ± 1000 bp of a RefSeq promoter. These stitched regions were ranked based on their H3K27ac signal, calculated as the product of peak length and signal density, with the control IgG input signal subtracted. SE were then defined using a geometric approach as those enhancers above the inflection point where the slope of the ranked signal curve equals one (y = x tangent). Bigwig files for display were made using RPM using bamCoverage 3.5.1. Heat maps for genome-wide occupancy were generated by deepTools 3.5.1 [42].

### Motif analysis

The genomic sequence within 100 bp of the summit of each CUT&Tag-seq peak was generated from the UCSC website with repeats masked with “N”. Motif analysis was performed by Hypergeometric Optimization of Motif EnRichment (HOMER) v4.11 [43].

### Cell growth assay

To assess the effects of knocking down each CRC gene on cell growth, RMS cells were seeded into 96-well plates at a density of 1000–1500 cells per well, and relative cell growth at days 1, 3, 5 and 7 was evaluated by CellTiter-Glo Luminescent Cell Viability Assay (Promega #G7571). To assess the effects of small-molecule inhibitors on cell growth, RMS cells were seeded into 96-well plates at a density of 1500 cells per well one day prior to treatment. Small-molecule inhibitors (Table S7), alone or in combination, were added at day 0. Relative cell growth at days 1, 2 and 3 was evaluated by CellTiter-Glo Luminescent Cell Viability Assay (Promega #G7571).

### The half-maximal inhibitory concentration (IC_50_) assay

RMS cells were seeded into a 96-well plate at a density of 1500 cells per well one day prior to treatment. After 3 days of treatment with vehicle (DMSO) or small-molecule inhibitors (Table S7) at various concentrations, relative cell growth was evaluated by CellTiter-Glo Luminescent Cell Viability Assay (Promega #G7571) as a percentage of the vehicle (DMSO) control. The IC_50_ value was determined with GraphPad Prism 9.

### Paraffin sectioning and immunohistochemistry

Xenograft tumors harvested from mice were formalin-fixed, paraffin-embedded, and sectioned. The sections were stained with hematoxylin & eosin (H&E) and subjected to immunohistochemistry using primary antibodies against Ki-67 (HUABIO #HA721115) and cleaved caspase-3 (Asp175) (Cell Signaling Technology #9664).

### Statistical analysis

Statistical analyses were performed using GraphPad Prism 10.1.2 (GraphPad Software). Data are presented as mean ± SEM or mean ± SD as indicated in each of the figure legends unless otherwise indicated. The statistical tests used and the number of biological replicates are specified in each figure legend. Variance was similar between the groups being compared. For comparisons involving more than two groups and two independent variables, a two-way ANOVA was performed, followed by Dunnett’s multiple comparisons test to compare each experimental group with the control group. Dunnett’s test inherently adjusts for multiple comparisons by controlling the family-wise error rate. A *P* value < 0.05 was considered statistically significant.

## Supplementary information

Supplementary Information

Original Data

Table S1

Table S2

Table S3

Table S4

Table S5

Table S6

Table S7

## Data Availability

The RNA-seq and CUT&Tag-seq data generated in this study are available from the Genome Sequence Archive [44] in the National Genomics Data Center [45], China National Center for Bioinformation/Beijing Institute of Genomics, Chinese Academy of Sciences, under accession numbers HRA012985 and HRA012996. The RNA-seq data for RMS cell lines RD, RH30 and PLA-802, RMS PDCs (ZJUCH-RMS-PDC-15, 21, 22, 39BC, 39AC, 40, 54, 77, 78BC) and the matched tumor tissues, as well as non-RMS PDCs derived from RMS tissues, were obtained from https://ngdc.cncb.ac.cn/gsa-human under accession numbers HRA007320 and HRA007324 [6].

## References

[1] Skapek SX, Ferrari A, Gupta AA, Lupo PJ, Butler E, Shipley J, et al. Rhabdomyosarcoma. Nat Rev Dis Primers. 2019;5:1. 30617281 10.1038/s41572-018-0051-2 PMC7456566

[2] WHO Classification of Tumors Editorial Board. Soft tissue and bone tumors. WHO Classification of Tumors, 5th Edition, 2020;3:201–13.

[3] Kim J, Light N, Subasri V, Young EL, Wegman-Ostrosky T, Barkauskas DA, et al. Pathogenic germline variants in cancer susceptibility genes in children and young adults with rhabdomyosarcoma. JCO Precis Oncol. 2021;5:PO.20.00218. 10.1200/PO.20.00218 PMC8169077 34095712

[4] Shern JF, Selfe J, Izquierdo E, Patidar R, Chou HC, Song YK, et al. Genomic classification and clinical outcome in rhabdomyosarcoma: a report from an international consortium. J Clin Oncol. 2021;39:2859–71. 34166060 10.1200/JCO.20.03060 PMC8425837

[5] Shern JF, Chen L, Chmielecki J, Wei JS, Patidar R, Rosenberg M, et al. Comprehensive genomic analysis of rhabdomyosarcoma reveals a landscape of alterations affecting a common genetic axis in fusion-positive and fusion-negative tumors. Cancer Discov. 2014;4:216–31. 24436047 10.1158/2159-8290.CD-13-0639 PMC4462130

[6] Hu Y, He Z, Liu S, Ying W, Chen Y, Zhao M, et al. Patient-derived rhabdomyosarcoma cells recapitulate the genetic and transcriptomic landscapes of primary tumors. iScience. 2024;27:110862. 39319271 10.1016/j.isci.2024.110862 PMC11417342

[7] Owosho AA, Chen S, Kashikar S, Zhang L, Chen CL, Wexler LH, et al. Clinical and molecular heterogeneity of head and neck spindle cell and sclerosing rhabdomyosarcoma. Oral Oncol. 2016;58:e6–e11. 27261172 10.1016/j.oraloncology.2016.05.009 PMC5518412

[8] Saoud C, Dermawan JK, Sharma AE, Tap W, Wexler LH, Antonescu CR. Genomic profiling of pleomorphic rhabdomyosarcoma reveals a genomic signature distinct from that of embryonal rhabdomyosarcoma. Genes Chromosomes Cancer. 2024;63.e23238. 38722224 10.1002/gcc.23238 PMC11664927

[9] Chen Y, Xu L, Lin RY, Muschen M, Koeffler HP. Core transcriptional regulatory circuitries in cancer. Oncogene. 2020;39:6633–46. 32943730 10.1038/s41388-020-01459-w PMC7581508

[10] Hnisz D, Abraham BJ, Lee TI, Lau A, Saint-Andre V, Sigova AA, et al. Super-enhancers in the control of cell identity and disease. Cell. 2013;155:934–47. 24119843 10.1016/j.cell.2013.09.053 PMC3841062

[11] Saint-Andre V, Federation AJ, Lin CY, Abraham BJ, Reddy J, Lee TI, et al. Models of human core transcriptional regulatory circuitries. Genome Res. 2016;26:385–96. 26843070 10.1101/gr.197590.115 PMC4772020

[12] Gryder BE, Pomella S, Sayers C, Wu XS, Song Y, Chiarella AM, et al. Histone hyperacetylation disrupts core gene regulatory architecture in rhabdomyosarcoma. Nat Genet. 2019;51:1714–22. 31784732 10.1038/s41588-019-0534-4 PMC6886578

[13] Gryder BE, Yohe ME, Chou HC, Zhang X, Marques J, Wachtel M, et al. PAX3-FOXO1 establishes myogenic super enhancers and confers BET bromodomain vulnerability. Cancer Discov. 2017;7:884–99. 28446439 10.1158/2159-8290.CD-16-1297 PMC7802885

[14] Lee JK, Liu Z, Sa JK, Shin S, Wang J, Bordyuh M, et al. Pharmacogenomic landscape of patient-derived tumor cells informs precision oncology therapy. Nat Genet. 2018;50:1399–411. 30262818 10.1038/s41588-018-0209-6 PMC8514738

[15] Xie Z, Bailey A, Kuleshov MV, Clarke DJB, Evangelista JE, Jenkins SL, et al. Gene set knowledge discovery with EnrichR. Curr Protoc. 2021;1:e90. 33780170 10.1002/cpz1.90 PMC8152575

[16] Hsu JY, Danis EP, Nance S, O’Brien JH, Gustafson AL, Wessells VM, et al. SIX1 reprograms myogenic transcription factors to maintain the rhabdomyosarcoma undifferentiated state. Cell Rep. 2022;38:110323. 35108532 10.1016/j.celrep.2022.110323 PMC8917510

[17] Szklarczyk D, Kirsch R, Koutrouli M, Nastou K, Mehryary F, Hachilif R, et al. The STRING database in 2023: protein–protein association networks and functional enrichment analyses for any sequenced genome of interest. Nucleic Acids Res. 2023;51:D638–D46. 36370105 10.1093/nar/gkac1000 PMC9825434

[18] Li T, Fu J, Zeng Z, Cohen D, Li J, Chen Q, et al. TIMER2.0 for analysis of tumor-infiltrating immune cells. Nucleic Acids Res. 2020;48:W509–W14. 32442275 10.1093/nar/gkaa407 PMC7319575

[19] Boyer LA, Lee TI, Cole MF, Johnstone SE, Levine SS, Zucker JP, et al. Core transcriptional regulatory circuitry in human embryonic stem cells. Cell. 2005;122:947–56. 16153702 10.1016/j.cell.2005.08.020 PMC3006442

[20] Boeva V, Louis-Brennetot C, Peltier A, Durand S, Pierre-Eugene C, Raynal V, et al. Heterogeneity of neuroblastoma cell identity defined by transcriptional circuitries. Nat Genet. 2017;49:1408–13. 28740262 10.1038/ng.3921

[21] Lin CY, Erkek S, Tong Y, Yin L, Federation AJ, Zapatka M, et al. Active medulloblastoma enhancers reveal subgroup-specific cellular origins. Nature. 2016;530:57–62. 26814967 10.1038/nature16546 PMC5168934

[22] Chen Y, Xu L, Mayakonda A, Huang ML, Kanojia D, Tan TZ, et al. Bromodomain and extraterminal proteins foster the core transcriptional regulatory programs and confer vulnerability in liposarcoma. Nat Commun. 2019;10:1353. 30903020 10.1038/s41467-019-09257-z PMC6430783

[23] Cancer Genome Atlas Research Network.Comprehensive and integrated genomic characterization of adult soft tissue sarcomas. Cell. 2017;171:950–965.e28. 29100075 10.1016/j.cell.2017.10.014 PMC5693358

[24] Selvamurugan N, Kwok S, Partridge NC. Smad3 interacts with JunB and Cbfa1/Runx2 for transforming growth factor-beta1-stimulated collagenase-3 expression in human breast cancer cells. J Biol Chem. 2004;279:27764–73. 15084595 10.1074/jbc.M312870200

[25] Tao T, Sondalle SB, Shi H, Zhu S, Perez-Atayde AR, Peng J, et al. The pre-rRNA processing factor DEF is rate-limiting for the pathogenesis of MYCN-driven neuroblastoma. Oncogene. 2017;36:3852–67. 28263972 10.1038/onc.2016.527 PMC5501763

[26] Tao T, Shi H, Mariani L, Abraham BJ, Durbin AD, Zimmerman MW, et al. LIN28B regulates transcription and potentiates MYCN-induced neuroblastoma through binding to ZNF143 at target gene promotors. Proc Natl Acad Sci USA. 2020;117:16516–26. 32601179 10.1073/pnas.1922692117 PMC7368283

[27] Dobin A, Davis CA, Schlesinger F, Drenkow J, Zaleski C, Jha S, et al. STAR: ultrafast universal RNA-seq aligner. Bioinformatics. 2013;29:15–21. 23104886 10.1093/bioinformatics/bts635 PMC3530905

[28] Liao Y, Smyth GK, Shi W. featureCounts: an efficient general-purpose program for assigning sequence reads to genomic features. Bioinformatics. 2014;30:923–30. 24227677 10.1093/bioinformatics/btt656

[29] Anders S, Huber W. Differential expression analysis for sequence count data. Genome Biol. 2010;11:R106. 20979621 10.1186/gb-2010-11-10-r106 PMC3218662

[30] Wang K, Li M, Hakonarson H. ANNOVAR: functional annotation of genetic variants from high-throughput sequencing data. Nucleic Acids Res. 2010;38:e164. 20601685 10.1093/nar/gkq603 PMC2938201

[31] Landrum MJ, Chitipiralla S, Brown GR, Chen C, Gu B, Hart J, et al. ClinVar: improvements to accessing data. Nucleic Acids Res. 2020;48:D835–D44. 31777943 10.1093/nar/gkz972 PMC6943040

[32] Li Q, Wang K. InterVar: Clinical interpretation of genetic variants by the 2015 ACMG-AMP guidelines. Am J Hum Genet. 2017;100:267–80. 28132688 10.1016/j.ajhg.2017.01.004 PMC5294755

[33] Tate JG, Bamford S, Jubb HC, Sondka Z, Beare DM, Bindal N, et al. COSMIC: the catalogue of somatic mutations in cancer. Nucleic Acids Res. 2019;47:D941–D47. 30371878 10.1093/nar/gky1015 PMC6323903

[34] Edmonson MN, Patel AN, Hedges DJ, Wang Z, Rampersaud E, Kesserwan CA, et al. Pediatric cancer variant pathogenicity information exchange (PeCanPIE): a cloud-based platform for curating and classifying germline variants. Genome Res. 2019;29:1555–65. 31439692 10.1101/gr.250357.119 PMC6724669

[35] Mayakonda A, Lin DC, Assenov Y, Plass C, Koeffler HP. Maftools: efficient and comprehensive analysis of somatic variants in cancer. Genome Res. 2018;28:1747–56. 30341162 10.1101/gr.239244.118 PMC6211645

[36] Subramanian A, Tamayo P, Mootha VK, Mukherjee S, Ebert BL, Gillette MA, et al. Gene set enrichment analysis: a knowledge-based approach for interpreting genome-wide expression profiles. Proc Natl Acad Sci USA. 2005;102:15545–50. 16199517 10.1073/pnas.0506580102 PMC1239896

[37] Langmead B, Salzberg SL. Fast gapped-read alignment with Bowtie 2. Nat Methods. 2012;9:357–9. 22388286 10.1038/nmeth.1923 PMC3322381

[38] Zhang Y, Liu T, Meyer CA, Eeckhoute J, Johnson DS, Bernstein BE, et al. Model-based analysis of ChIP-Seq (MACS). Genome Biol. 2008;9:R137. 18798982 10.1186/gb-2008-9-9-r137 PMC2592715

[39] Yu G, Wang LG, He QY. ChIPseeker: an R/Bioconductor package for ChIP peak annotation, comparison and visualization. Bioinformatics. 2015;31:2382–3. 25765347 10.1093/bioinformatics/btv145

[40] Durbin AD, Zimmerman MW, Dharia NV, Abraham BJ, Iniguez AB, Weichert-Leahey N, et al. Selective gene dependencies in MYCN-amplified neuroblastoma include the core transcriptional regulatory circuitry. Nat Genet. 2018;50:1240–46. 30127528 10.1038/s41588-018-0191-z PMC6386470

[41] Whyte WA, Orlando DA, Hnisz D, Abraham BJ, Lin CY, Kagey MH, et al. Master transcription factors and mediator establish super-enhancers at key cell identity genes. Cell. 2013;153:307–19. 23582322 10.1016/j.cell.2013.03.035 PMC3653129

[42] Ramirez F, Ryan DP, Gruning B, Bhardwaj V, Kilpert F, Richter AS, et al. deepTools2: a next-generation web server for deep-sequencing data analysis. Nucleic Acids Res. 2016;44:W160–5. 27079975 10.1093/nar/gkw257 PMC4987876

[43] Duttke SH, Chang MW, Heinz S, Benner C. Identification and dynamic quantification of regulatory elements using total RNA. Genome Res. 2019;29:1836–46. 31649059 10.1101/gr.253492.119 PMC6836739

[44] Chen T, Chen X, Zhang S, Zhu J, Tang B, Wang A, et al. The genome sequence archive family: toward explosive data growth and diverse data types. Genom Proteom Bioinform. 2021;19:578–83. 10.1016/j.gpb.2021.08.001 PMC9039563 34400360

[45] CNCB-NGDC Members and Partners. Database Resources of the National Genomics Data Center, China National Center for Bioinformation in 2024. Nucleic Acids Res. 2024;52:D18–D32. 38018256 10.1093/nar/gkad1078 PMC10767964

