# Supplementary material for: Core transcriptional regulatory circuitry identified from patient-derived tumor cells provides a specific vulnerability for rhabdomyosarcoma
Source: Cell Death Dis. 2026 Jun 13;17(1):744. doi: 10.1038/s41419-026-08949-x (PMC13490588; doi:10.1038/s41419-026-08949-x)
Supplement: Supplementary file 1 [file 41419_2026_8949_MOESM1_ESM.pdf]

Supplementary Information

Core transcriptional regulatory circuitry identified from patient-derived tumor cells provides a specific vulnerability for rhabdomyosarcoma

Shuangai Liu[1,2,3], Ziqi He[1,2], Xue Chen[1,2,4], Yinbing Tang[1,2], Min He[1,2], Lifeng Zhang[1,2], Weili Xu[3,7], Jinhu Wang[1,2,5,6,7], Ting Tao[1,2,5,6,7]

[1]Department of Surgical Oncology, Children's Hospital Zhejiang University School of Medicine, National Clinical Research Center for Children and Adolescents' Health and Diseases, Hangzhou 310052, China.

[2]Pediatric Cancer Research Center, National Clinical Research Center for Children and Adolescents' Health and Diseases, Children's Hospital Zhejiang University School of Medicine, Hangzhou 310052, China.

[3]Department of Pediatric Surgery, The Second Hospital of Hebei Medical University, Shijiazhuang, 050000, China.

[4]The First Clinical Institute, Zunyi Medical University, Zunyi 563000, China.

[5]Zhejiang Key Laboratory of Neonatal Diseases, Hangzhou 310052, China.

[6]Cancer Center, Zhejiang University, Hangzhou 310058, China.

[7]These authors contributed equally: Weili Xu, Jinhu Wang, Ting Tao.

Correspondence: Weili Xu; Jinhu Wang; Ting Tao. E-mail: 27300526@hebmu.edu.cn; wjh@zju.edu.cn; taot@zju.edu.cn

SUPPLEMENTARY FIGURES

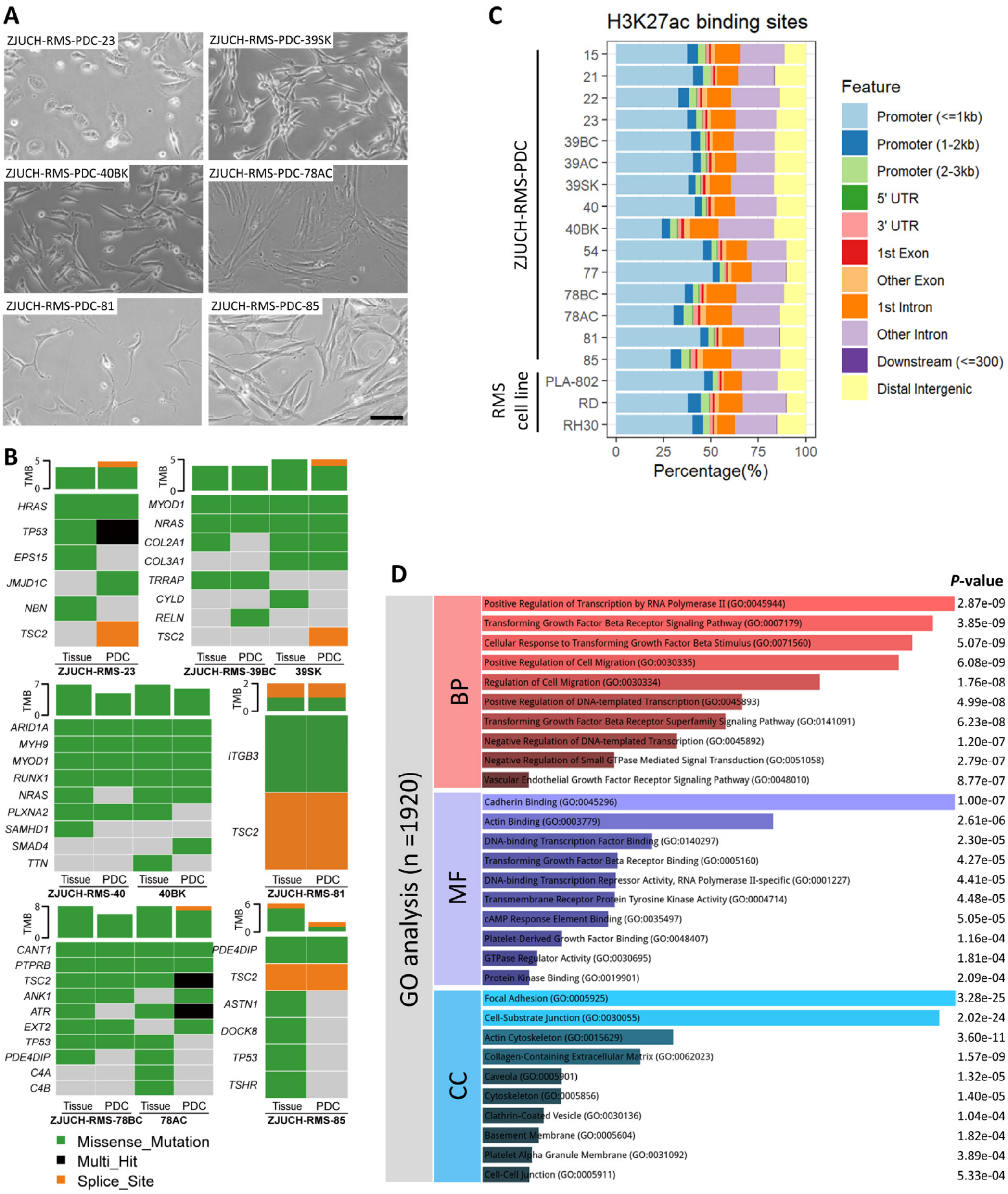

Figure S1 RMS PDCs recapitulate the genetic and transcriptomic landscapes of primary tumors. A Morphologies of 6 RMS PDCs identified in this study. Scale bar, 100 μm. **B** Oncoplots comparing high-confidence variants between RMS PDCs and matched primary tumors. TMB, tumor mutation burden. **C** Genome-wide distribution of H3K27ac peaks (*P* < 1e-9) across a panel of RMS PDCs (n = 15) and cell lines (n = 3). **D** Gene ontology (GO) analysis of 1920 SE-associated genes identified from RMS PDCs and cell lines using the Enrichr program. BP, biological process; MF, molecular function; CC, cellular component. The top 10 terms ranked by *P*-value are shown.

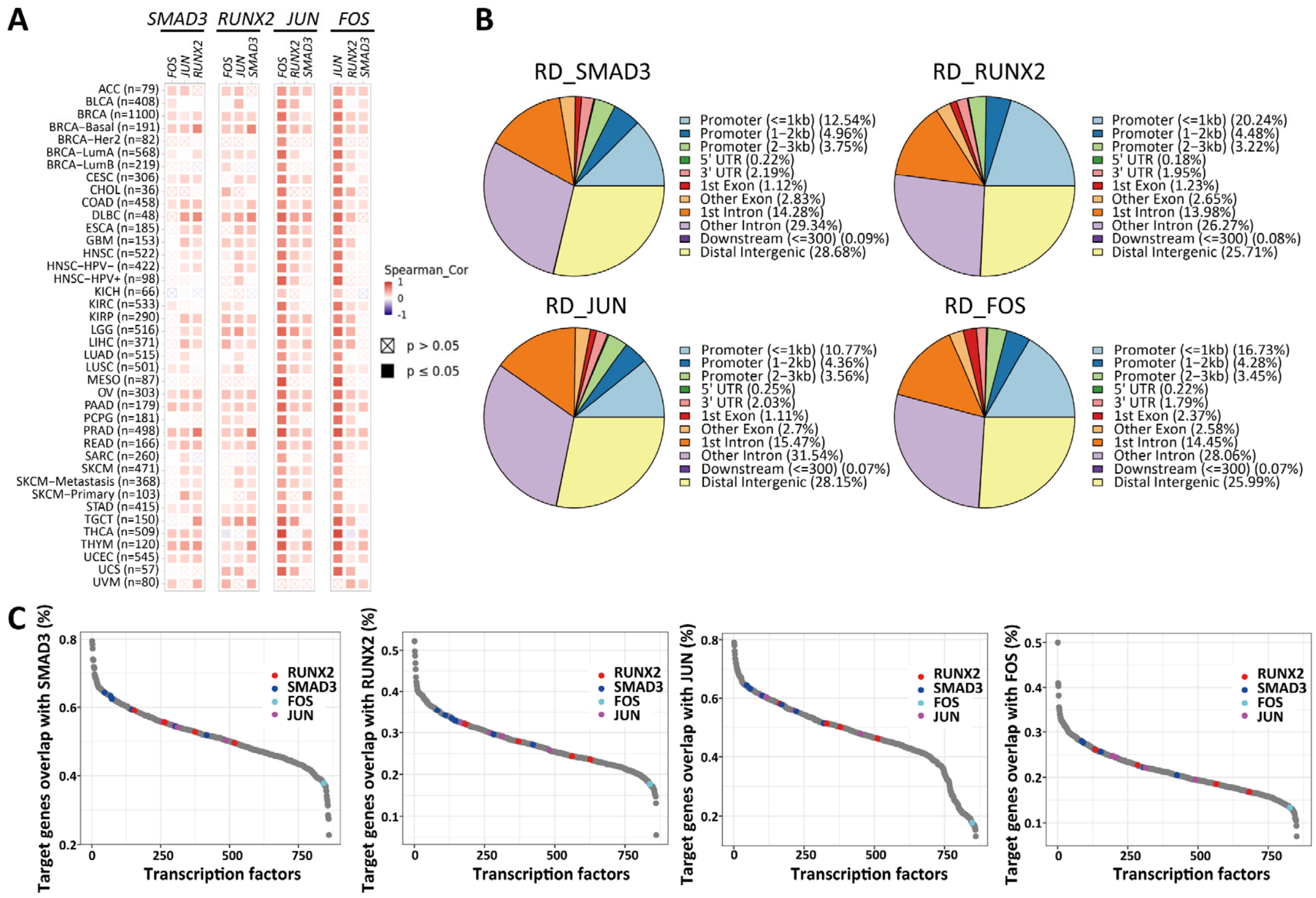

Figure S2 Genome-wide distributions of *SMAD3*, *RUNX2*, *JUN* and *FOS* in rhabdomyosarcoma. A Correlation analysis of RNA expression among *SMAD3*, *RUNX2*, *JUN* and *FOS* across multiple tumor types using the TIMER2.0 program. Data were obtained from The Cancer Genome Atlas (TCGA). Heatmap gives the purity-adjusted partial spearman's rho value as the degree of their correlation. Tumor types are annotated using TCGA study abbreviations, with the number of cases for each type shown in parentheses. **B** Annotation of SMAD3, RUNX2, JUN and FOS CUT&Tag-seq peaks identified in RD cells. **C** The percentage of target genes of known transcription factors overlapping with those of SMAD3, RUNX2, JUN and FOS in RD cells. The analysis was performed using the ChEA 2022 and ENCODE and ChEA Consensus transcription factors databases via the Enrichr program, and data were plotted using the ggplot2 in R.

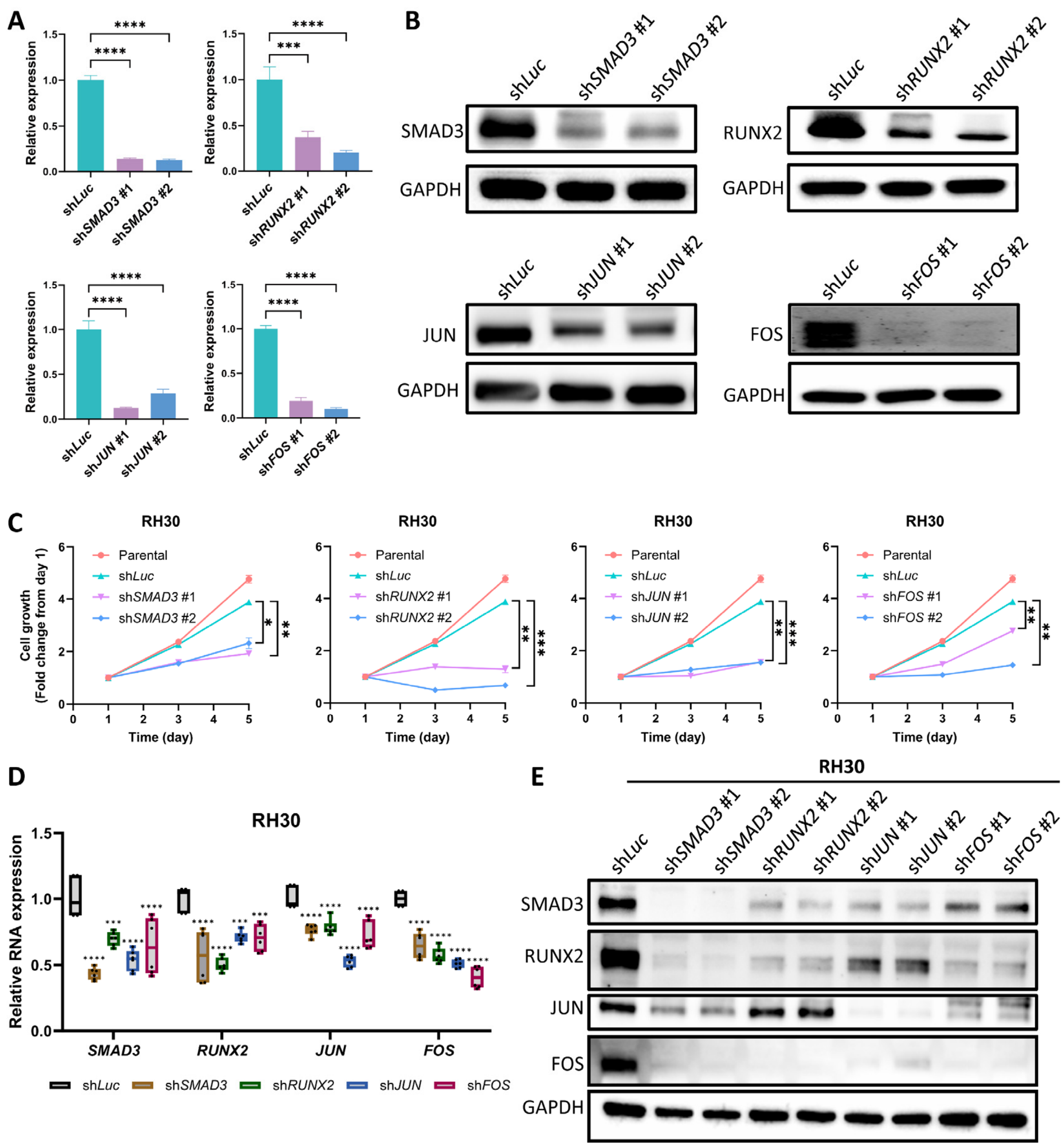

Figure S3 Knockdown of each CRC member inhibited RMS cell growth and decreased expression of the other CRC components. A, B RD cells were transfected with control sh*Luc* or with two independent shRNAs targeting *SMAD3*, *RUNX2*, *JUN* or *FOS*. **A** The mRNA expression levels of *SMAD3*, *RUNX2*, *JUN* and *FOS* were detected by quantitative RT-PCR. Values were normalized to *GAPDH* and represented the means ± SD of triplicate experiments. Values were compared with one-way ANOVA by Dunnett's test. ***P < 0.001, ****P < 0.0001. **B** The protein levels of SMAD3, RUNX2, JUN and FOS were detected by western blot analysis; GAPDH was used as a loading control. **C-E** RH30 cells were untransfected (Parental), or

transfected with control sh*Luc* or with two independent shRNAs targeting *SMAD3*, *RUNX2*, *JUN* or *FOS*. **C** Relative cell growth at days 1, 3 and 5 was evaluated. Values are means ± SEM of triplicate experiments. Mean values were compared with two-way ANOVA by Dunnett's test. **P* < 0.05, ***P* < 0.01, ****P* < 0.001. **D** The mRNA expression levels of *SMAD3*, *RUNX2*, *JUN* and *FOS* were detected by quantitative RT-PCR. Values were normalized to *GAPDH*, boxes indicate the median (horizontal line), 25th percentile and 75th percentile; Whiskers indicate the minimum to the maximum. Values were compared with one-way ANOVA by Dunnett's test. ****P* < 0.001, *****P* < 0.0001. **E** The protein levels of SMAD3, RUNX2, JUN and FOS were detected by western blot analysis; GAPDH was used as a loading control.

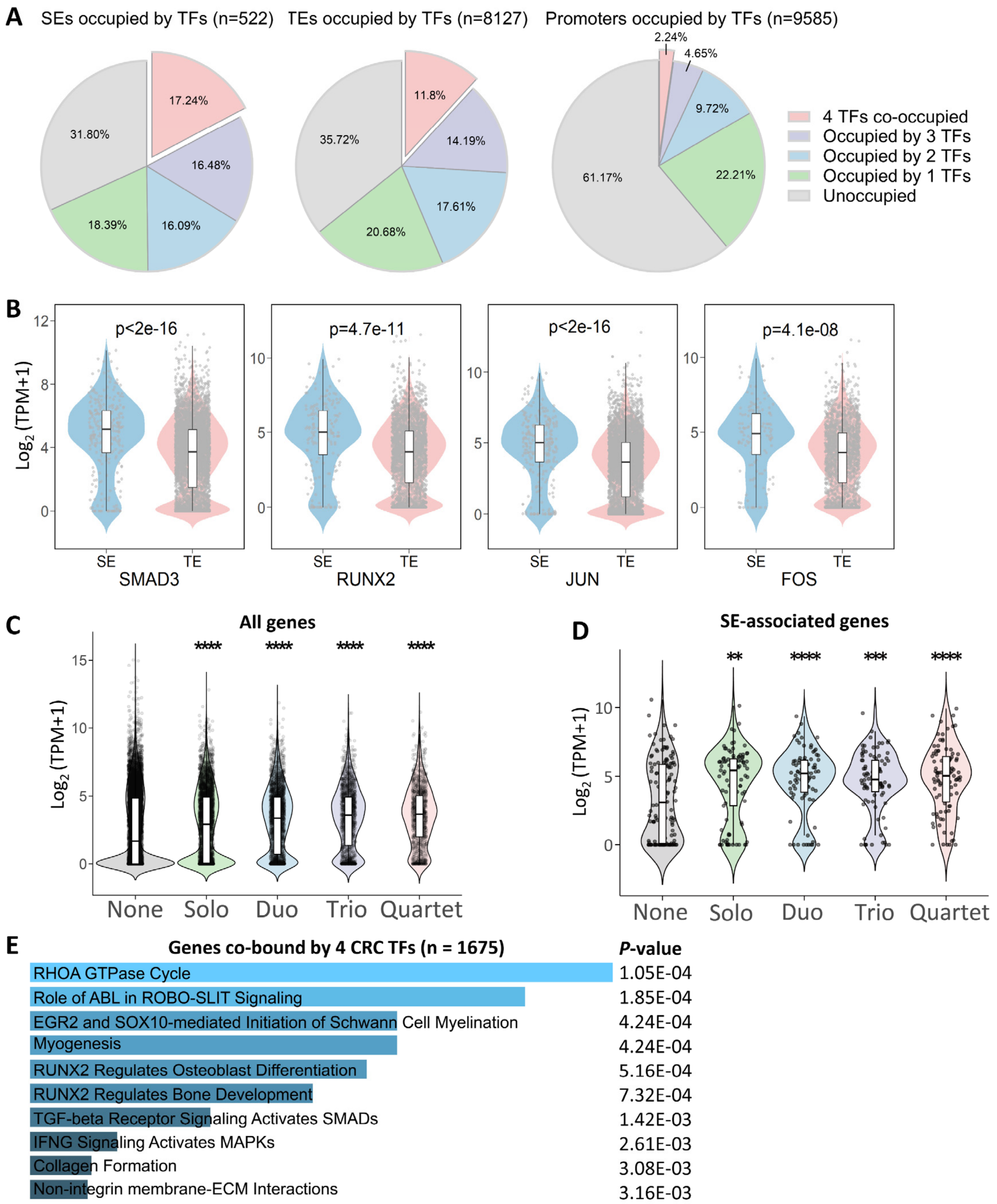

Figure S4 Genes associated with CRC members expressed at significantly high levels. A Pie charts showing the distribution of SEs, TEs, and promoters according to the number of bound CRC factors in RD cells. **B** Violin plots showing mRNA expression levels of genes bound by each CRC member, stratified by enhancer activity status in RD cells. Boxes indicate the median (horizontal line), 25th percentile and 75th percentile; Whiskers indicate distances from the largest and smallest value to each end of the box that are within 1.5× box length. Values are $\log_2(\text{TPM}+1)$, which were

compared with Welch's t test. TPM, transcripts per kilobase of exon per million mapped reads; SE, super-enhancer; TE, typical-enhancer. **C, D** Violin plots showing mRNA expression levels of **C** all genes or **D** super-enhancer (SE)-associated genes, stratified by different CRC member binding combinations in RD cells. Boxes indicate the median (horizontal line), 25th percentile and 75th percentile; Whiskers indicate distances from the largest and smallest value to each end of the box that are within 1.5× box length. Values are $\log_2$(TPM+1), which were compared with the "None" group by Welch's t test. ***P* < 0.01, ****P* < 0.001, *****P* < 0.0001. The labels None, Solo, Duo, Trio, and Quartet represent the number of CRC members occupying a given gene. TPM, transcripts per kilobase of exon per million mapped reads. **E** Gene ontology analysis of 1675 genes co-bound by the four CRC transcription factors (TFs) in RD cells using the Enrichr program. The top 10 terms ranked by *P*-value are shown.

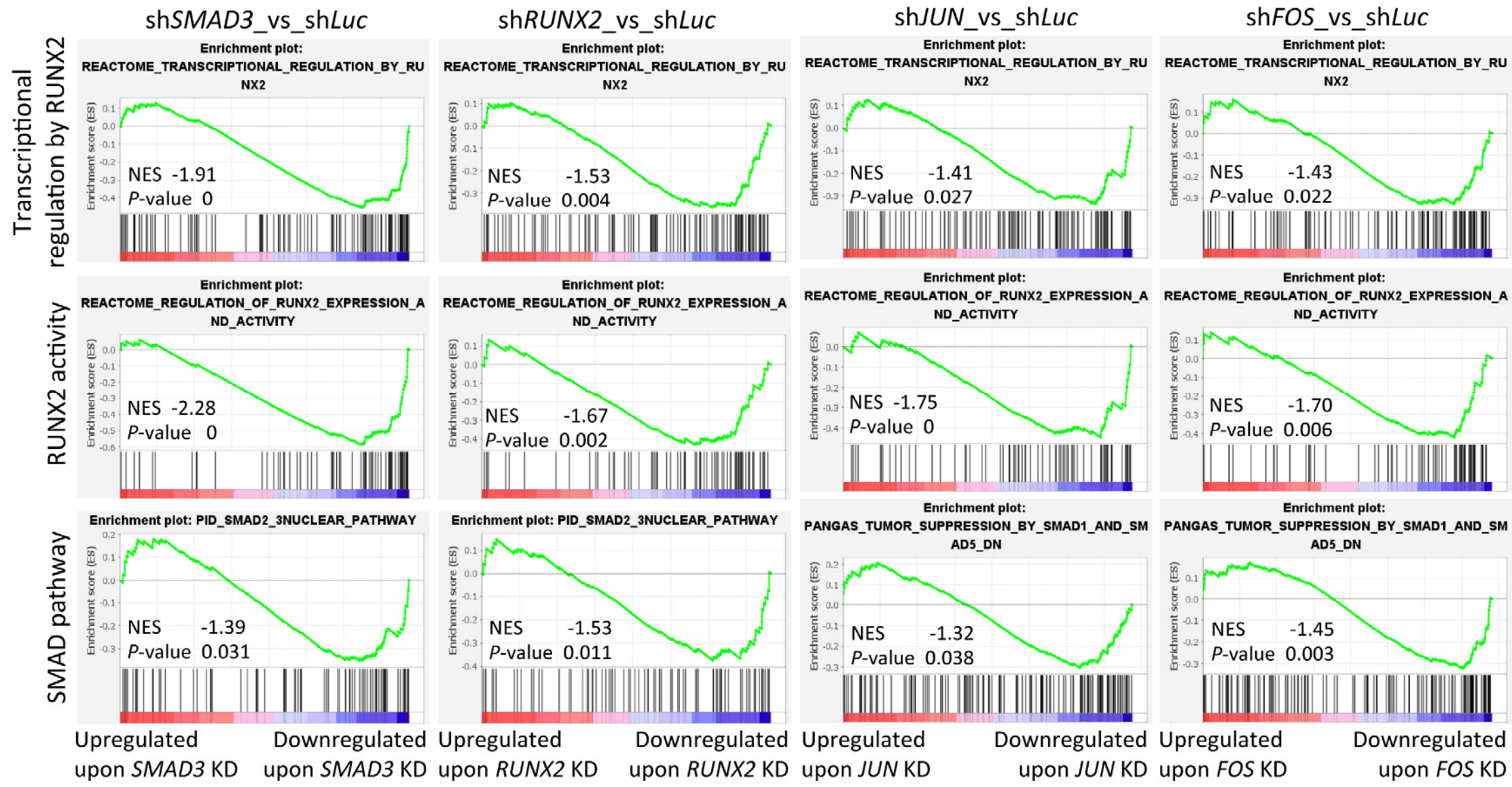

Figure S5 GSEA of gene expression profiles upon knockdown of each CRC member. Representative plots of significantly enriched gene signatures indicating the repression of RUNX and SMAD pathways upon knockdown each of the CRC members in RD cells. Genes are ranked by score and plotted along the x axis as vertical black bars. NES, normalized enrichment score; KD, knockdown.

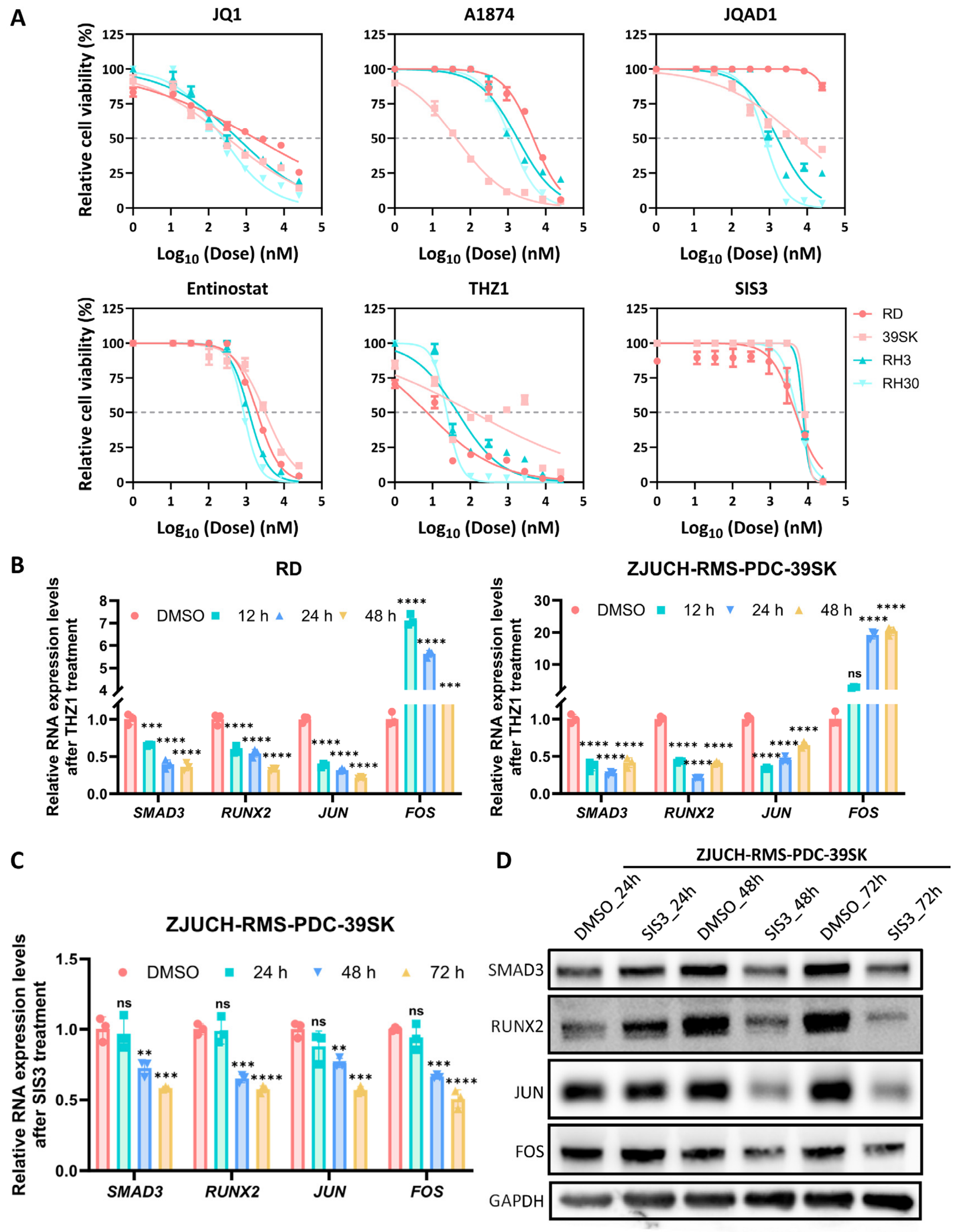

Figure S6 Agents targeting the epigenetic program inhibit rhabdomyosarcoma cell growth. A Cell viability (% relative to DMSO-treated cells, y value) of RMS cells was measured after 72 hours of treatment with various concentrations (0 to 25,000 nM, x value) of agents targeting epigenetic program, and half-maximal inhibitory concentration (IC_{50}) values were calculated with the "log(inhibitor) vs. normalized

response - variable slope" model. Values are means ± SD of triplicate experiments. **B** RD or ZJUCH-RMS-PDC-39SK cells were treated with vehicle (DMSO) or THZ1 (7 nM for RD, 122 nM for ZJUCH-RMS-PDC-39SK) for 12, 24 or 48 hours. The mRNA expression levels of *SMAD3*, *RUNX2*, *JUN* and *FOS* were detected by quantitative RT-PCR. Values were normalized to *GAPDH* and represented the means ± SD of triplicate experiments. Values were compared with one-way ANOVA by Dunnett's test. ***$P < 0.001$, ****$P < 0.0001$. ns, not significant. **C, D** ZJUCH-RMS-PDC-39SK cells were treated with vehicle (DMSO) or SIS3 (8.26 μM) for 24, 48 or 72 hours. **C** The mRNA expression levels of *SMAD3*, *RUNX2*, *JUN* and *FOS* were detected by quantitative RT-PCR. Values were normalized to *GAPDH* and represented the means ± SD of triplicate experiments. Values were compared with one-way ANOVA by Dunnett's test. **$P < 0.01$, ***$P < 0.001$, ****$P < 0.0001$. ns, not significant. **D** The protein levels of SMAD3, RUNX2, JUN and FOS were detected by western blot analysis; GAPDH was used as a loading control.

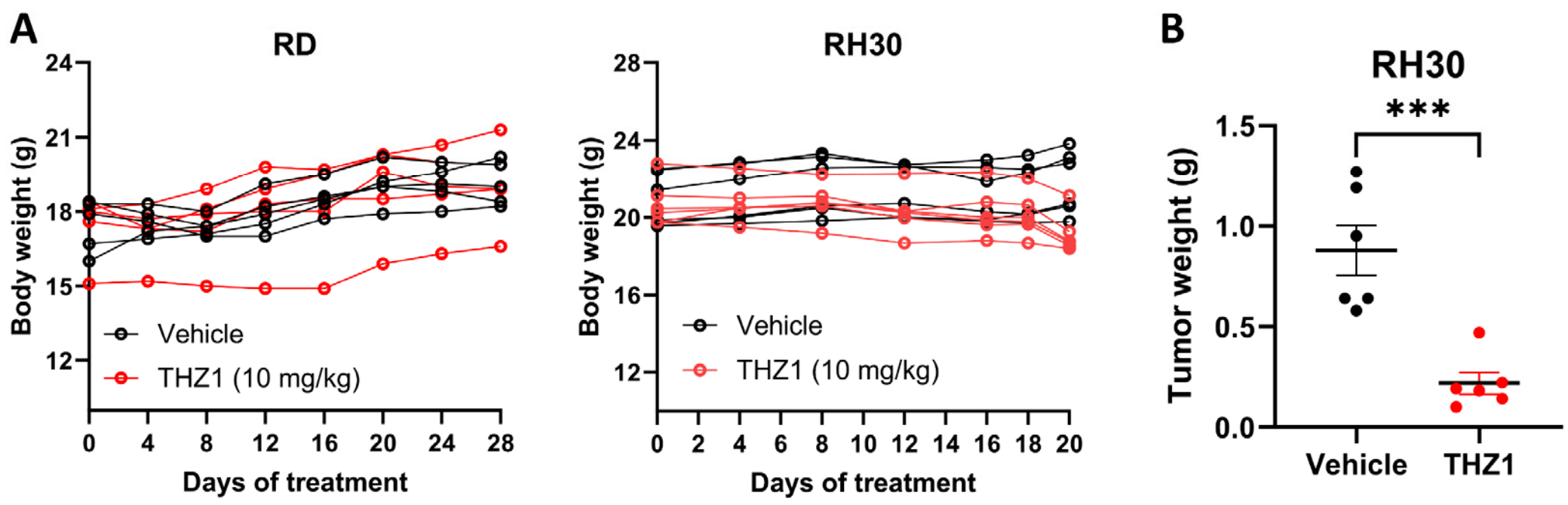

Figure S7 Mouse body weight and xenograft tumor weight following THZ1 treatment. **A, B** Mice were injected subcutaneously with RD or RH30 cells. Upon tumor establishment, vehicle (DMSO) or THZ1 (10 mg/kg) was administered twice daily starting from day 0. **A** Mice body weight was determined every 4 days thereafter. **B** At the experimental endpoint, the weight of RH30 xenograft tumors was determined. Each dot represents the tumor weight from an individual mouse. Horizontal bars indicate the means ± SEM, and statistical comparison was performed using a two-tailed unpaired *t*-test. ***$P < 0.001$.

SUPPLEMENTARY TABLES

Table S1. Summary of RMS PDCs used in this study.

Table S2. High-confidence variants identified from RNA-seq in RMS PDCs and matched primary tumors.

Table S3. Super-enhancer-associated transcription factors identified in RMS PDCs and cell lines.

Table S4. CRC components inferred by CRCMapper across 18 RMS samples.

Table S5. Expression of core transcription factors across multiple panels of RMS samples.

Values are log_2 (TPM+1) of RNA-seq data.

Table S6. All oligonucleotide sequences used in this study.

Table S7. Small-molecule inhibitors used in this study.
