# Supplementary material for: Core transcriptional regulatory circuitry identified from patient-derived tumor cells provides a specific vulnerability for rhabdomyosarcoma
Source: Cell Death Dis. 2026 Jun 13;17(1):744. doi: 10.1038/s41419-026-08949-x (PMC13490588; doi:10.1038/s41419-026-08949-x)
Supplement: Supplementary file 2 [file 41419_2026_8949_MOESM2_ESM.pdf]

Supplementary Information

Core transcriptional regulatory circuitry identified from patient-derived tumor cells provides a specific vulnerability for rhabdomyosarcoma

Shuangai Liu[1,2,3], Ziqi He[1,2], Xue Chen[1,2,4], Yinbing Tang[1,2], Min He[1,2], Lifeng Zhang[1,2], Weili Xu[3,7], Jinhu Wang[1,2,5,6,7], Ting Tao[1,2,5,6,7]

[1]Department of Surgical Oncology, Children's Hospital Zhejiang University School of Medicine, National Clinical Research Center for Children and Adolescents' Health and Diseases, Hangzhou 310052, China.

[2]Pediatric Cancer Research Center, National Clinical Research Center for Children and Adolescents' Health and Diseases, Children's Hospital Zhejiang University School of Medicine, Hangzhou 310052, China.

[3]Department of Pediatric Surgery, The Second Hospital of Hebei Medical University, Shijiazhuang, 050000, China.

[4]The First Clinical Institute, Zunyi Medical University, Zunyi 563000, China.

[5]Zhejiang Key Laboratory of Neonatal Diseases, Hangzhou 310052, China.

[6]Cancer Center, Zhejiang University, Hangzhou 310058, China.

[7]These authors contributed equally: Weili Xu, Jinhu Wang, Ting Tao.

Correspondence: Weili Xu; Jinhu Wang; Ting Tao. E-mail: 27300526@hebmu.edu.cn; wjh@zju.edu.cn; taot@zju.edu.cn

SUPPLEMENTARY FIGURES

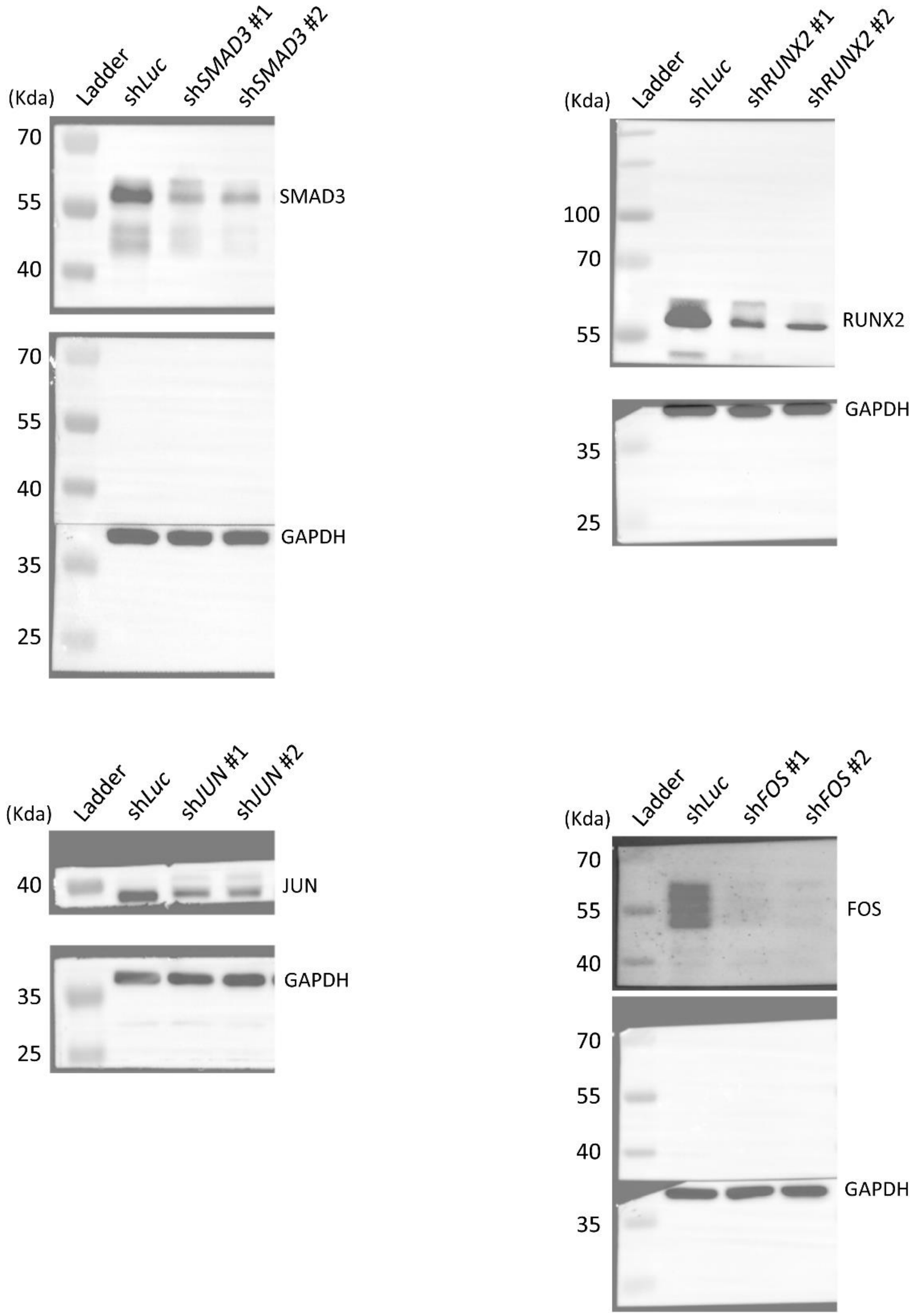

Figure S8 Full western blot results with lane markers. Full western blot results are shown for Fig. S3B. The positions of molecular weight markers are indicated.

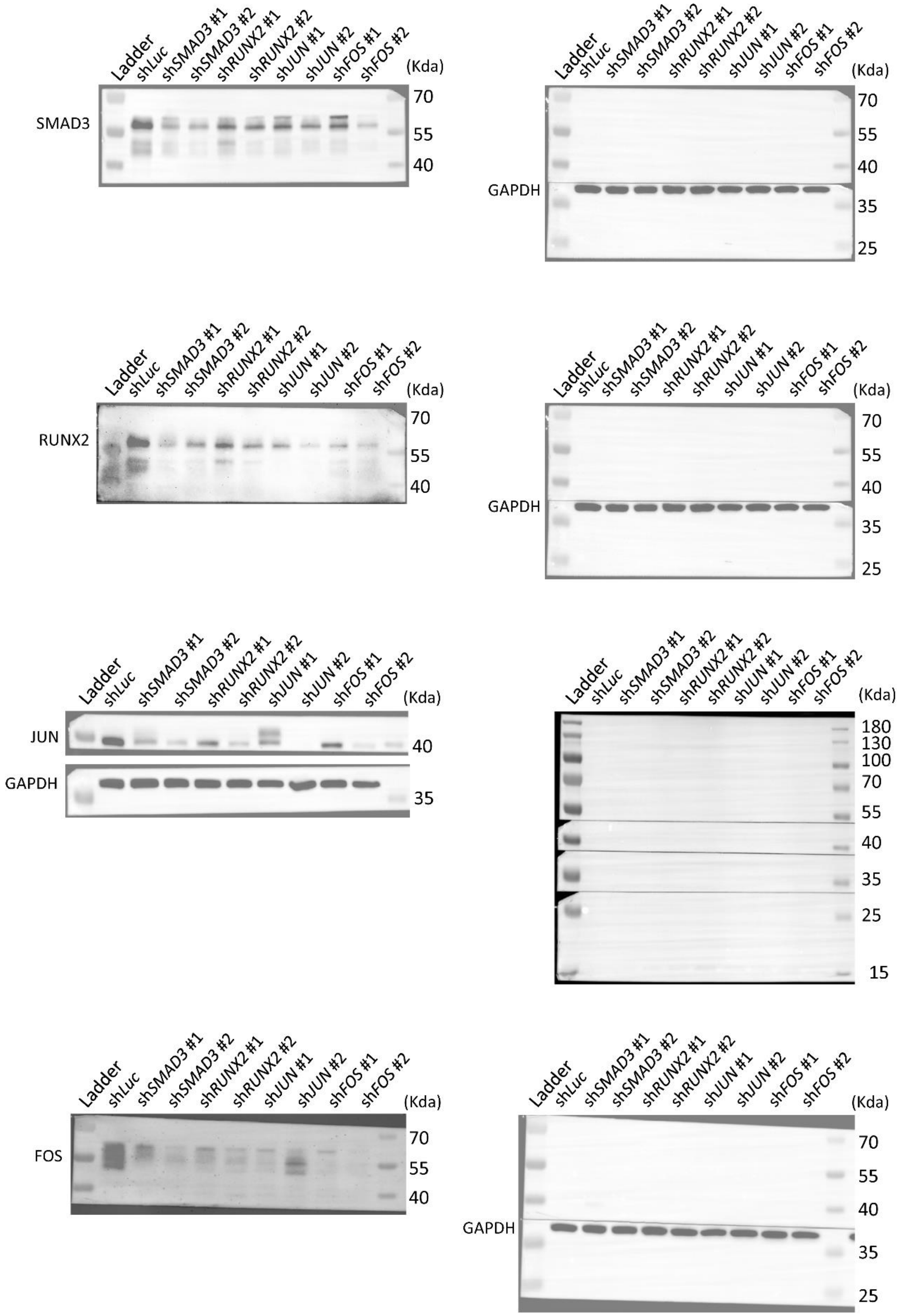

Figure S9 Full western blot results with lane markers. Full western blot results are shown for Fig. 3C. The positions of molecular weight markers are indicated.

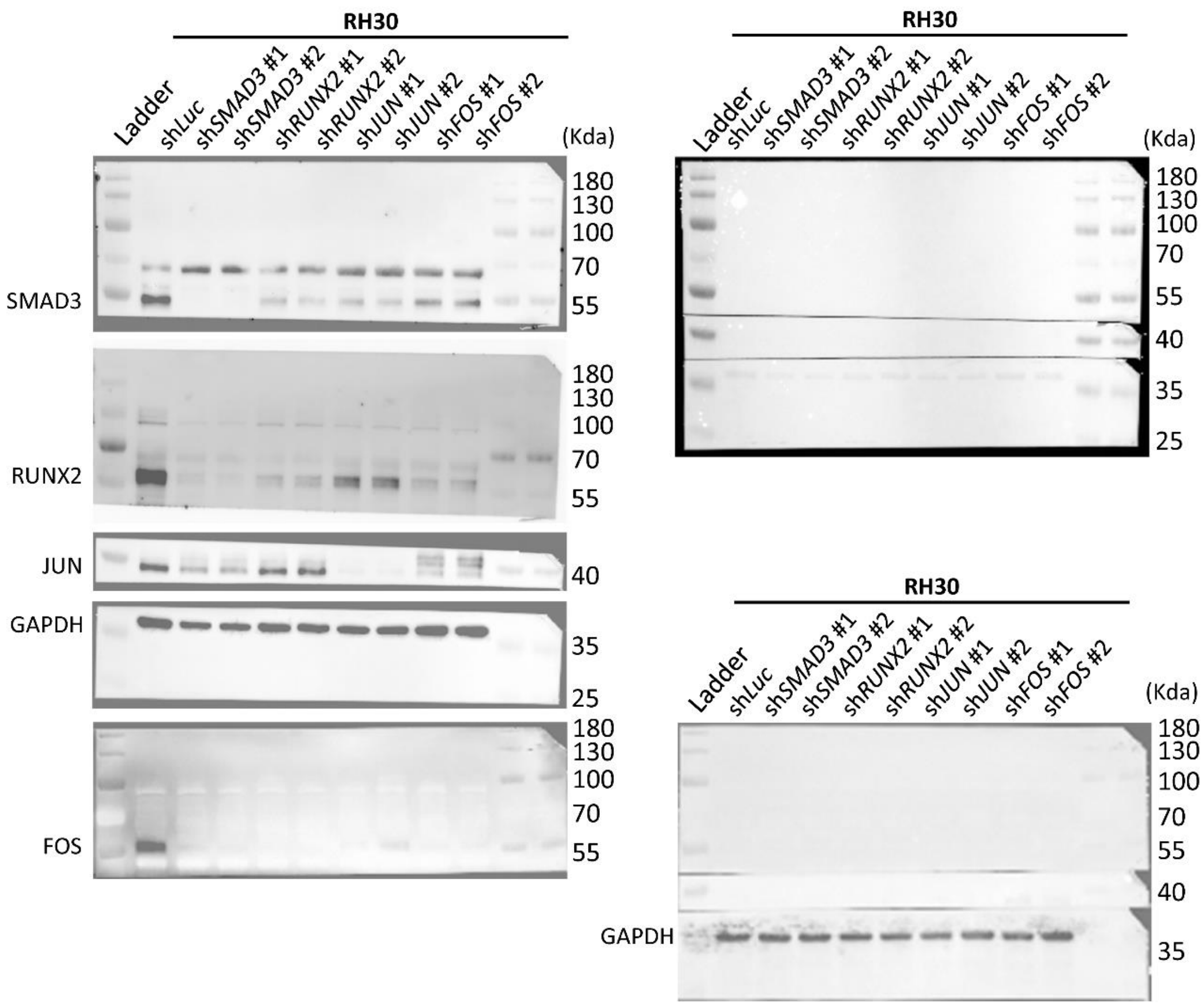

Figure S10 Full western blot results with lane markers. Full western blot results are shown for Fig. S3E. The positions of molecular weight markers are indicated.

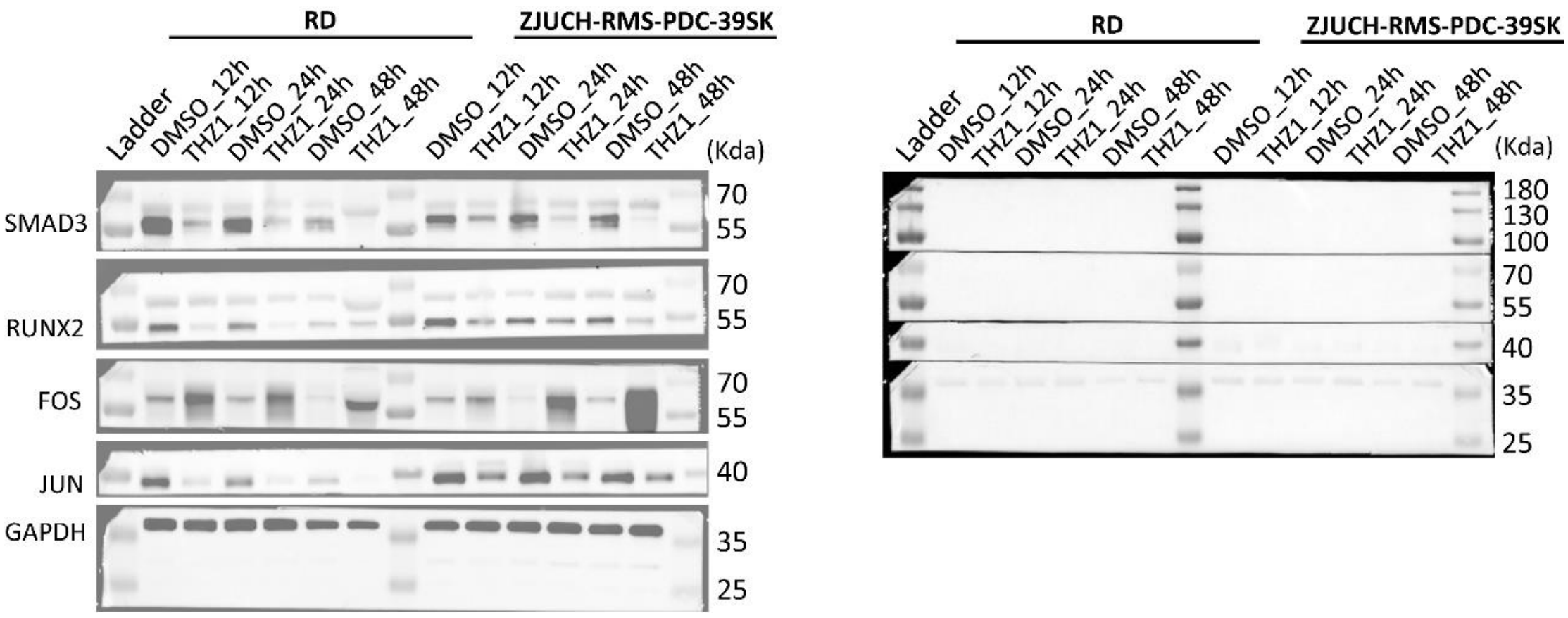

Figure S11 Full western blot results with lane markers. Full western blot results are shown for Fig. 4B. The positions of molecular weight markers are indicated.

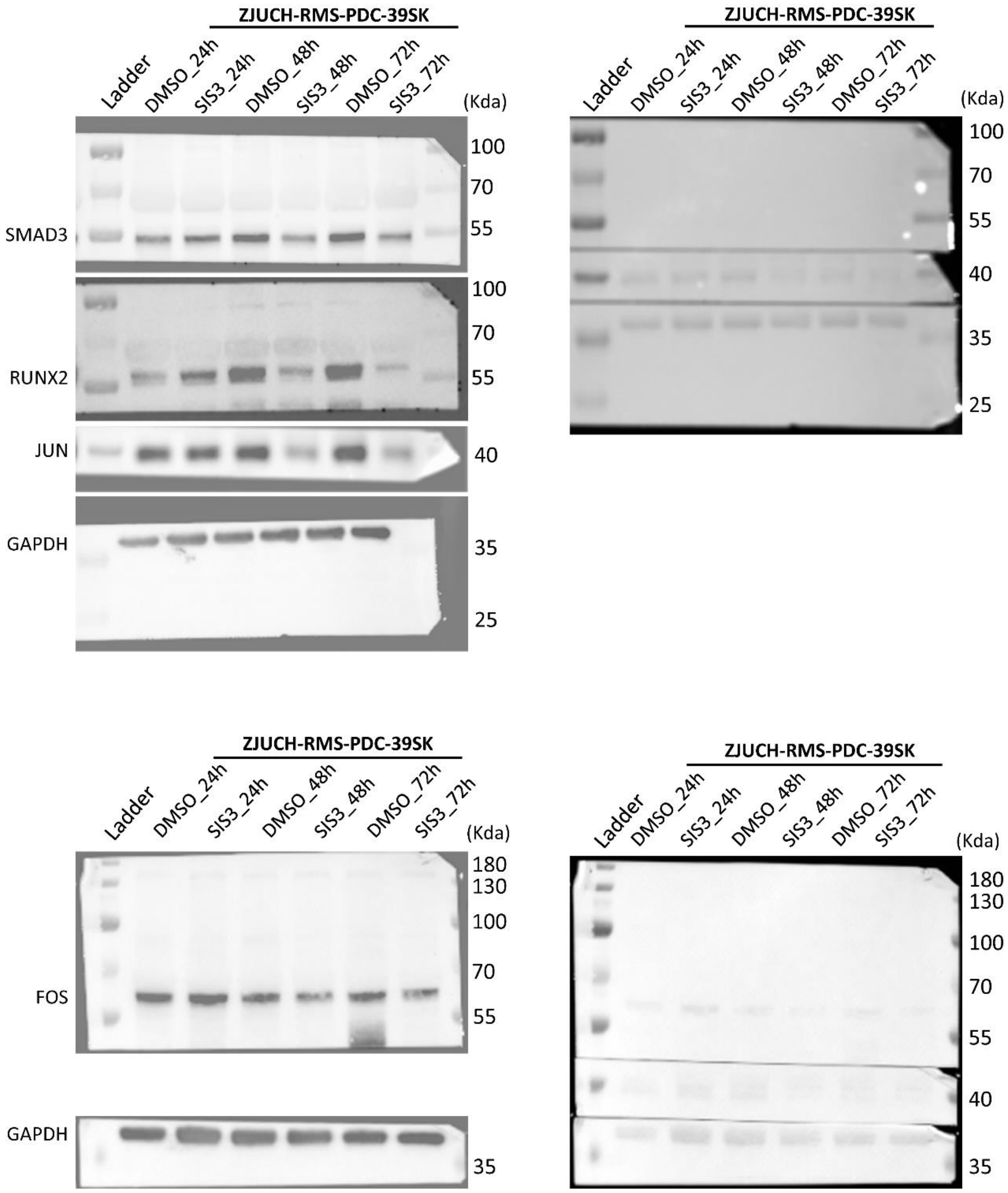

Figure S12 Full western blot results with lane markers. Full western blot results are shown for Fig. S6D. The positions of molecular weight markers are indicated.
